# Supplementary material for: De novo design of knotted tandem repeat proteins
Source: Nat Commun. 2023 Oct 24;14:6746. doi: 10.1038/s41467-023-42388-y (PMC10598012; doi:10.1038/s41467-023-42388-y)
Supplement: Supplementary file 1 — Supplementary Information [file 41467_2023_42388_MOESM1_ESM.pdf]

Supplementary Table S1. Diagrams, sequences and alignments of designed kcTRP proteins

1. Original kcTRP3 'trefoil' designs

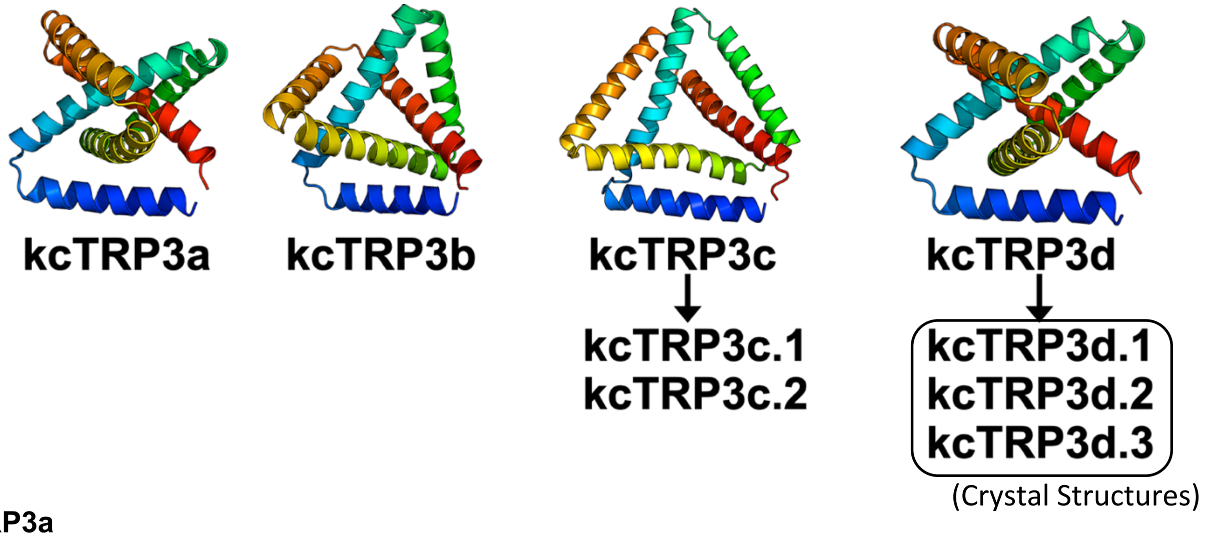

kcTRP3a

ATGGCTAGCAGCCATCATCATCATCATCATAGCAGCGGCCTGGTGCCGCGCGGCAGCTCCATGGGTAGCGACGATGAGCGTAAGCGTATCGACGA
GGAAGTGAAACGTCTGCAGGAAGAGCTGAAGAACAAAAGCCCGGATCAAATCCGTAAGGAGCTGAAAGAATGGATTCTGCGTATGTACGTGCTGTT
CCAGACCCTGGAGAAGAAACTGAGCGATGACGAGCGTAAGCGTATTGACGAGGAAGTTAAGCGCCTGCAGGAAGAGCTGAAAAATAAGAGCCCGG
ACCAGATTCGCAAAGAACTGAAGGAGTGGATTCTGCGTATGTATGTGCTGTTTCAAACTTTAGAGAAGAAACTGAGCGACGACGAGCGCAAACGCAT
CGACGAGGAAGTTAAGCGATTACAGGAAGAGCTGAAAAACAAGAGCCCGGACCAGATCCGCAAAGAACTGAAGGAATGGATCCTGCGCATGTACG
TTCTGTTCCAGACCCTGGAAAAGAAACTGAGCTAA

MASSHHHHHHSSGLVPRGSSMGSDDERKRIDEEVKRLQEELKNKSPDQIRKELKEWILRMYVLFQTLEKKLSDDERKRIDEEVKRLQEELKNKSPDQIRKE
LKEWILRMYVLFQTLEKKLSDDERKRIDEEVKRLQEELKNKSPDQIRKELKEWILRMYVLFQTLEKKLS*

kcTRP3b

ATGGCTAGCAGCCATCATCATCATCATCATAGCAGCGGCCTGGTGCCGCGCGGCAGCTCCATGGGTCCGGAGGAACTGGAGCGTGAACTGCGTGA
GGCGGAGGAAGACTACCGTAAGGCGAGCCCGGAGGAACAGAAACGTATCAAGAAAGCGCTGCTGGTGCTGGCGCGTGAGTACCTGCGTCAGCAA
CGTGAAAAGTATGGCCCGGAAGAGCTGGAACGCGAACTGCGTGAAGCGGAGGAAGATTATCGCAAAGCGAGCCCGGAGGAACAAAAACGTATTAA
GAAAGCGCTGCTGGTTCTGGCGCGCGAATACCTGCGTCAACAGCGTGAAAAATATGGCCCGGAAGAACTGGAACGCGAGCTGCGCGAAGCGGAG
GAAGACTATCGCAAGGCGAGCCCGGAAGAGCAGAAACGTATCAAGAAAGCGTTACTGGTTCTGGCGCGCGAGTACCTGCGTCAACAGCGCGAAAA
GTATGGCTAA

MASSHHHHHHSSGLVPRGSSMGPEELERELREAEEDYRKASPEEQKRIKKALLVLAREYLRQQREKYGPEELERELREAEEDYRKASPEEQKRIKKALLV
LAREYLRQQREKYGPEELERELREAEEDYRKASPEEQKRIKKALLVLAREYLRQQREKYG*

kcTRP3c

ATGGCTAGCAGCCATCATCATCATCATCATAGCAGCGGCCTGGTGCCGCGCGGCAGCTCCATGGGTAACCCGGAGCAGATCGAGAAGGAAATTAA
AGAGCGTGAGCGTAAGATCCGTGAAAAAATTAAGAAAGCGGGCATCAGCCGTGAGCAACTGGAAAAGCTGCGTGAGCTGTACCTGCGTCTGGAACT
GGCGCGTATTGCGGAGGAACTGCTGCGTAACGGTAAAAATCCGGAACAAATTGAAAAAGAGATCAAGGAACGCGAGCGCAAAATTCGCGAGAAGAT
TAAGAAAGCGGGTATTAGCCGCGAACAGCTGGAGAAACTGCGTGAGCTGTATCTGCGTCTGGAGCTGGCGCGCATCGCGGAAGAGCTGCTGCGCA
ATGGTAAAAATCCGGAACAGATTGAAAAAGAGATCAAAGAACGCGAGCGCAAAATCCGCGAGAAAATTAAGAAAGCGGGTATCAGCCGCGAACAGC
TGGAAAAACTGCGCGAACTGTACCTGCGTTTAGAGTTAGCGCGTATCGCGGAAGAACTGCTGCGCAACGGTAAAAACTAA

MASSHHHHHHSSGLVPRGSSMGNPEQIEKEIKERERKIREKIKKAGISREQLEKLRELYLRLELARIAEELLRNGKNPEQIEKEIKERERKIREKIKKAGISREQ
LEKLRELYLRLELARIAEELLRNGKNPEQIEKEIKERERKIREKIKKAGISREQLEKLRELYLRLELARIAEELLRNGKN*

kcTRP3d

ATGGCTAGCAGCCATCATCATCATCATCATAGCAGCGGCCTGGTGCCGCGCGGCAGCTCCATGGGTAGCGACGAACAGCGTCGTGAGCTGGAGGA
AAAAATCAAGAAAAAGCTGGAGGAACTGAAAACCAAGAGCGAGGAAGAGCGTAAAGAAATCAAGCTGCGTGTGATTGCGTACGTGCTGGTTCAGCT
GGAGGACCTGCAGAAGAACCTGAGCGATGAACAACGTCGTGAGCTGGAAGAGAAGATTAAAAAGAAACTGGAAGAGCTGAAAACCAAGAGCGAAG
AGGAACGCAAGGAAATCAAGCTGCGTGTTATTGCGTATGTTCTGGTGCAACTGGAAGACCTGCAGAAGAACTTAAGCGATGAGCAACGTCGTGAAC
TGGAAGAAAAGATCAAGAAAAAGCTGGAAGAACTGAAGACCAAAAGCGAGGAAGAGCGCAAGGAGATTAAACTGCGCGTTATCGCGTATGTTCTGG
TTCAGCTGGAGGATCTGCAAAAGAACCTGAGCTAA

MASSHHHHHHSSGLVPRGSSMGSDEQRRELEEKIKKKLEELKTKSEEERKEIKLRVIAYVLVQLEDLQKNLSDEQRRELEEKIKKKLEELKTKSEEERKEIKL
RVIAYVLVQLEDLQKNLSDEQRRELEEKIKKKLEELKTKSEEERKEIKLRVIAYVLVQLEDLQKNLS*

2. Alignments between original kcTRP3 'trefoil' designs

```
kcTRP3a:     Repeats = Helix 1(20 residues)- turn - Helix 2(25 residues)
kcTRP3b:     Repeats = Helix 1(17 residues)- turn - Helix 2(26 residues)
kcTRP3c:     Repeats = Helix 1(22 residues)- turn - Helix 2(26 residues)
kcTRP3d:     Repeats = Helix 1(20 residues)- turn - Helix 2(25 residues)
```

Alignment of full-length constructs:

```
kcTRP3a      MASSHHHHHHSSGLVPRGSSMGSDDERKRIDEEVKRLQEELKNKSPDQIR--KELKEWIL     58
kcTRP3b      MASSHHHHHHSSGLVPRGSSMGPE-EL---ERELREAEEDYRKASPEEQKRIKKALLVLA     56
kcTRP3c      MASSHHHHHHSSGLVPRGSSMGNPEQI---EKEIKERERKIREKIKKAG-ISREQLEKLR     56
kcTRP3d      MASSHHHHHHSSGLVPRGSSMGSDEQRRELEEKIKKKLEELKTKSEEERK--EIKLRVIA     58
                 (His-tag + thrombin site)

kcTRP3b      REYLRQ---------QREKYGPEELERELREAEEDYRKASPEEQKRIKKALLVLAREYLR     107
kcTRP3c      ELYLRLELARIAEELLRNGKNPEQIEKEIKERERKIREKIKKAG-ISREQLEKLRELYLR     115
kcTRP3d      YVLVQLED--LQKNLS--DEQRRELEEKIKKKLEELKTKSEEERK--EIKLRVIAYVLVQ     112
kcTRP3a      RMYVLFQT--LEKKLS--DDERKRIDEEVKRLQEELKNKSPDQIR--KELKEWILRMYVL     112

kcTRP3a      FQT--LEKKLS--DDERKRIDEEVKRLQEELKNKSPDQIR--KELKEWILRMYVLFQT--     164
kcTRP3b      Q---------QREKYGPEELERELREAEEDYRKASPEEQKRIKKALLVLAREYLRQQREK     158
kcTRP3c      LELARIAEELLRNGKNPEQIEKEIKERERKIREKIKKAG-ISREQLEKLRELYLRLELAR     174
kcTRP3d      LED--LQKNLS--DEQRRELEEKIKKKLEELKTKSEEERK--EIKLRVIAYVLVQLED--     164

kcTRP3a      LEKKLS-----   170
kcTRP3b      YG---------   160
kcTRP3c      IAEELLRNGKN   185
kcTRP3d      LQKNLS-----   170
```

Alignment of individual repeats:

```
kcTRP3a      DDERKRIDEEVKRLQEELKNKSPDQIRKELKE-WILRMYV--LFQTLEKKLS--------     49
kcTRP3b      ---PEELERELREAEEDYRKASPE------EQKRIKK-----ALLVLAREYLRQQREKYG     46
kcTRP3c      ---PEQIEKEIKERERKIREKIKKAGISREQLEKLRELYLRLELARIAEELLRNGKN---     54
kcTRP3d      DEQRRELEEKIKKKLEELKTKSEEER-KEIKLRVIAYVLV--QLEDLQKNLS--------     49
                 ..::.:::.  .. :    .      :   :        :  : .:
```

Alignment of individual repeats in kcTRP3a and kcTRP3d:

```
kcTRP3a      MASSHHHHHHSSGLVPRGSSMGSDDERKRIDEEVKRLQEELKNKSPDQIRKELKEWILRM     60
kcTRP3d      MASSHHHHHHSSGLVPRGSSMGSDEQRRELEEKIKKKLEELKTKSEEERKEIKLRVIAYV     60
             ************************::*:.::*::*:  ****.** :: ::   . *  :

kcTRP3a      YVLFQTLEKKLSDDERKRIDEEVKRLQEELKNKSPDQIRKELKEWILRMYVLFQTLEKKL     120
kcTRP3d      LVQLEDLQKNLSDEQRRELEEKIKKKLEELKTKSEEERKEIKLRVIAYVLVQLEDLQKNL     120
              * :: *:*:***::*:.::*::*:  ****.** :: ::   . *  : * :: *:*:*
```

3. Second generation kcTRP3 ‘trefoil’ designs

kcTRP3c.1

ATGGCTAGCAGCCATCATCATCATCATCATAGCAGCGGCCTGGTGCCGCGCGGCAGCTCCATGGGCAACCCGGAACTGATTGAACTGGAAATTAAA
GCAATCGAACGTGCTATTCGCGAAAAAATTAAGAAAGCGGGTATCAGCCGCGAACAGCTGGAAAAACTGCGTGAACTGTATCTGCGCCTGGAACTG
GCGCGTATTGCCGAAGAACTGCTGCGTAACGGCAAAAATCCGGAACTGATCGAACTGGAAATTAAAGCGATCGAACGTGCCATCCGTGAGAAGATT
AAGAAAGCGGGTATTTCTCGCGAACAACTGGAAAAACTGCGCGAACTGTACCTGCGTCTGGAACTGGCACGCATTGCTGAAGAACTGCTGCGCAAT
GGTAAAAACCCTGAGCTGATTGAACTGGAAATCAAAGCCATTGAACGCGCCATCCGTGAGAAAATTAAGAAAGCGGGCATTAGTCGTGAGCAATTAG
AGAAGCTGCGCGAGTTATATCTGCGCTTAGAGCTGGCGCGTATCGCGGAAGAACTGCTGCGCAACGGCAAAAATTAA

MASSHHHHHHSSGLVPRGSSMGNPELIELEIKAIERAIREKIKKAGISREQLEKLRELYLRLELARIAEELLRNGKNPELIELEIKAIERAIREKIKKAGISREQLE
KLRELYLRLELARIAEELLRNGKNPELIELEIKAIERAIREKIKKAGISREQLEKLRELYLRLELARIAEELLRNGKN

kcTRP3c.2

ATGGCTAGCAGCCATCATCATCATCATCATAGCAGCGGCCTGGTGCCGCGCGGCAGCTCCATGGGCAACCCGGAACAGATTGAAAAAGAAATCAAA
GAACGTGAACGCAAAATTCGCGAAAAAATCCTGAAAGCAGGTATTAGCCGTGAACAACTGGAAAAACTGCGTGAACTGTATCTGCGCCTGGAACTG
GCACGTATCGCTGAAGAACTGCTGCGCAACGGCAAAAATCCGGAACAAATCGAAAAAGAAATTAAAGAACGCGAACGTAAAATCCGTGAAAAAATCG
CGAAAGCCGGTATTTCTCGCGAACAGCTGGAAAAACTGCGCGAACTGTACCTGCGTCTGGAACTGGCGCGCATCGCCGAAGAACTGCTGCGTAAT
GGTAAAAATCCGGAACAAATTGAGAAGGAAATTAAAGAACGTGAACGTAAAATCCGCGAAAAAATTTGGAAAGCCGGTATTAGTCGCGAGCAGTTAG
AGAAACTGCGCGAGTTATATCTGCGCTTAGAGCTGGCACGTATTGCCGAAGAACTGCTGCGCAATGGCAAAAATTAA

MASSHHHHHHSSGLVPRGSSMGNPEQIEKEIKERERKIREKILKAGISREQLEKLRELYLRLELARIAEELLRNGKNPEQIEKEIKERERKIREKIAKAGISREQ
LEKLRELYLRLELARIAEELLRNGKNPEQIEKEIKERERKIREKIWKAGISREQLEKLRELYLRLELARIAEELLRNGKN

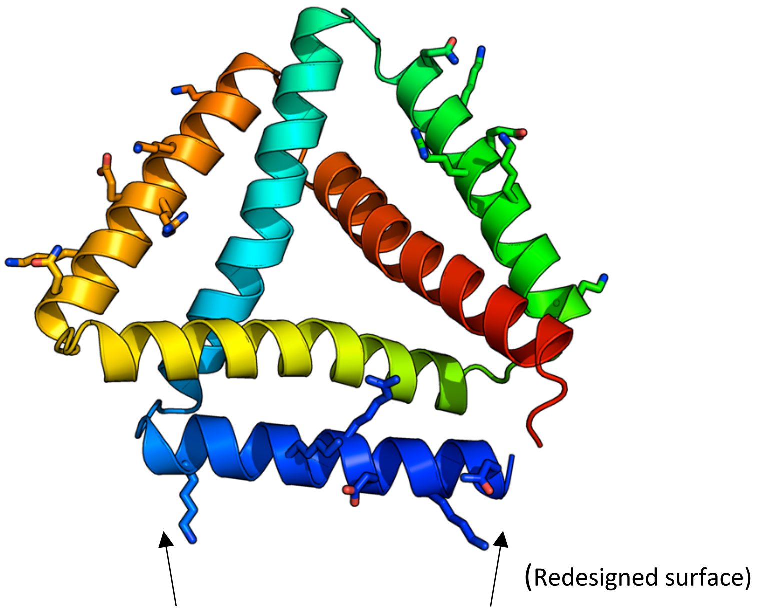


```
kcTRP3c        MASSHHHHHHSSGLVPRGSSMGNPEQIEKEIKERERKIREKIKKAGISREQLEKLRELYL   60
kcTRP3c.1      MASSHHHHHHSSGLVPRGSSMGNPELIELEIKAIERAIREKIKKAGISREQLEKLRELYL   60
kcTRP3c.2      MASSHHHHHHSSGLVPRGSSMGNPEQIEKEIKERERKIREKILKAGISREQLEKLRELYL   60
               ************************* ** ***  ** ***** *****************
                   (His-tag + thrombin site)

kcTRP3c        RLELARIAEELLRNGKNPEQIEKEIKERERKIREKIKKAGISREQLEKLRELYLRLELAR   120
kcTRP3c.1      RLELARIAEELLRNGKNPELIELEIKAIERAIREKIKKAGISREQLEKLRELYLRLELAR   120
kcTRP3c.2      RLELARIAEELLRNGKNPEQIEKEIKERERKIREKIAKAGISREQLEKLRELYLRLELAR   120
               ******************* ** ***  ** ***** ***********************

kcTRP3c        IAEELLRNGKNPEQIEKEIKERERKIREKIKKAGISREQLEKLRELYLRLELARIAEELL   180
kcTRP3c.1      IAEELLRNGKNPELIELEIKAIERAIREKIKKAGISREQLEKLRELYLRLELARIAEELL   180
kcTRP3c.2      IAEELLRNGKNPEQIEKEIKERERKIREKIWKAGISREQLEKLRELYLRLELARIAEELL   180
               ************* ** ***  ** ***** *****************************

kcTRP3c        RNGKN    185
kcTRP3c.1      RNGKN    185
kcTRP3c.2      RNGKN    185
               *****
```

kcTRP3d.1

ATGGCTAGCAGCCATCATCATCATCATCATAGCAGCGGCCTGGTGCCGCGCGGCAGCTCCATGGGCAGCGATGAACAGCGTCGCGAACTGGAAGA
AAAAATCAAATTCAAACTGGCGGAACTGGCCAGCAAATCTGAAGAAGAACGTAAAGAAATCAAACTGCGCGTGATCGCATATGTGCTGGTTCAGCTG
GAAGATCTGCAGAAAAACCTGTCTGACGAACAACGTCGCGAGTTAGAAGAAAAAATTAAATTTAAACTGGCAGAACTGGCTAGTAAATCCGAAGAAG
AACGCAAAGAAATTAAACTGCGTGTTATCGCTTACGTCCTGGTGCAATTAGAGGATCTGCAGAAAAACTTATCCGACGAACAACGTCGCGAATTAGA
GGAAAAAATTAAATTCAAACTGGCCGAACTGGCCTCAAAATCGGAAGAAGAACGAAAGGAAATCAAACTGCGCGTCATCGCATATGTTCTGGTCCAA
TTAGAAGATCTGCAGAAAAACTTAAGCTAA

MASSHHHHHHSSGLVPRGSSMGSDEQRRELEEKIKFKLAELASKSEEERKEIKLRVIAYVLVQLEDLQKNLSDEQRRELEEKIKFKLAELASKSEEERKEIKL
RVIAYVLVQLEDLQKNLSDEQRRELEEKIKFKLAELASKSEEERKEIKLRVIAYVLVQLEDLQKNLS*

kcTRP3d.2

ATGGCTAGCAGCCATCATCATCATCATCATAGCAGCGGCCTGGTGCCGCGCGGCAGCTCCATGGGCAGCGATGAACAGCGTCGCGAACTGGAAGA
AAAAATTAAATGGAAACTGGCGGAACTGGCCAGCAAATCTGAAGAAGAACGTAAAGAAATCAAACTGCGCGTGATCGCATATGTGCTGGTTCAGCTG
GAAGATCTGCAGAAAAACCTGTCTGACGAACAACGTCGCGAGTTAGAAGAAAAAATCAAATGGAAACTGGCAGAACTGGCTAGTAAATCCGAAGAAG
AACGCAAAGAAATTAAACTGCGTGTTATCGCTTACGTCCTGGTGCAATTAGAGGATCTGCAGAAAAACTTATCCGACGAACAACGTCGCGAATTAGA
GGAGAAGATCAAATGGAAACTGGCCGAACTGGCCTCAAAATCGGAAGAAGAACGAAAGGAAATCAAACTGCGCGTCATCGCATATGTTCTGGTCCA
ATTAGAAGATCTGCAGAAAAACTTAAGCTAA

MASSHHHHHHSSGLVPRGSSMGSDEQRRELEEKIKWKLAELASKSEEERKEIKLRVIAYVLVQLEDLQKNLSDEQRRELEEKIKWKLAELASKSEEERKEIK
LRVIAYVLVQLEDLQKNLSDEQRRELEEKIKWKLAELASKSEEERKEIKLRVIAYVLVQLEDLQKNLS*

kcTRP3d.3

ATGGCTAGCAGCCATCATCATCATCATCATAGCAGCGGCCTGGTGCCGCGCGGCAGCTCCATGGGCAGCGATGAACAGCGTCGCGAACTGGAAGA
AAAAATCAAACTGAAACTGGAAGAACTGAAAACCAAATCTGAAGAAGAACGTAAAGAAATCAAACTGCGCGTGATCGCATATGTGCTGGTTCAGCTG
GAAGATCTGCAGAAAAACCTGAGTGACGAACAACGTCGCGAGTTAGAAGAAAAAATCAAAGCCAAGTTAGAAGAACTGAAAACGAAATCCGAAGAAG
AACGCAAAGAAATTAAACTGCGTGTTATCGCCTACGTCCTGGTGCAATTAGAGGATCTGCAGAAAAACTTATCAGACGAACAACGTCGCGAATTAGA
GGAAAAAATCAAATGGAAGTTAGAGGAGCTGAAAACCAAATCGGAAGAAGAACGAAAGGAAATCAAACTGCGCGTCATCGCGTATGTTCTGGTCCAA
TTAGAAGATCTGCAGAAAAACTTAAGCTAA

MASSHHHHHHSSGLVPRGSSMGSDEQRRELEEKIKLKLEELKTKSEEERKEIKLRVIAYVLVQLEDLQKNLSDEQRRELEEKIKAKLEELKTKSEEERKEIKL
RVIAYVLVQLEDLQKNLSDEQRRELEEKIKWKLEELKTKSEEERKEIKLRVIAYVLVQLEDLQKNLS*

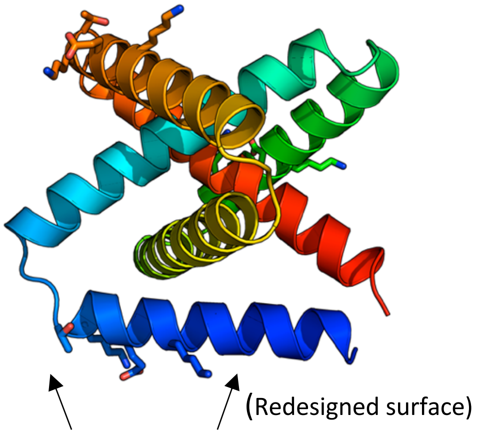


```
kcTRP3d       MASSHHHHHHSSGLVPRGSSMGSDEQRRELEEKIKKKLEELKTKSEEERKEIKLRVIAYV     60
kcTRP3d.1     MASSHHHHHHSSGLVPRGSSMGSDEQRRELEEKIKFKLAELASKSEEERKEIKLRVIAYV     60
kcTRP3d.2     MASSHHHHHHSSGLVPRGSSMGSDEQRRELEEKIKWKLAELASKSEEERKEIKLRVIAYV     60
kcTRP3d.3     MASSHHHHHHSSGLVPRGSSMGSDEQRRELEEKIKLKLEELKTKSEEERKEIKLRVIAYV     60
              *********************************** ** ** :*****************
                 (His-tag + thrombin site)

kcTRP3d       LVQLEDLQKNLSDEQRRELEEKIKKKLEELKTKSEEERKEIKLRVIAYVLVQLEDLQKNL     120
kcTRP3d.1     LVQLEDLQKNLSDEQRRELEEKIKFKLAELASKSEEERKEIKLRVIAYVLVQLEDLQKNL     120
kcTRP3d.2     LVQLEDLQKNLSDEQRRELEEKIKWKLAELASKSEEERKEIKLRVIAYVLVQLEDLQKNL     120
kcTRP3d.3     LVQLEDLQKNLSDEQRRELEEKIKAKLEELKTKSEEERKEIKLRVIAYVLVQLEDLQKNL     120
              ************************ ** ** :****************************

kcTRP3d       SDEQRRELEEKIKKKLEELKTKSEEERKEIKLRVIAYVLVQLEDLQKNLS  170
kcTRP3d.1     SDEQRRELEEKIKFKLAELASKSEEERKEIKLRVIAYVLVQLEDLQKNLS  170
kcTRP3d.2     SDEQRRELEEKIKWKLAELASKSEEERKEIKLRVIAYVLVQLEDLQKNLS  170
kcTRP3d.3     SDEQRRELEEKIKWKLEELKTKSEEERKEIKLRVIAYVLVQLEDLQKNLS  170
              ************* ** ** :*****************************
```

4. kcTRP5 ‘pentafoil’ designs

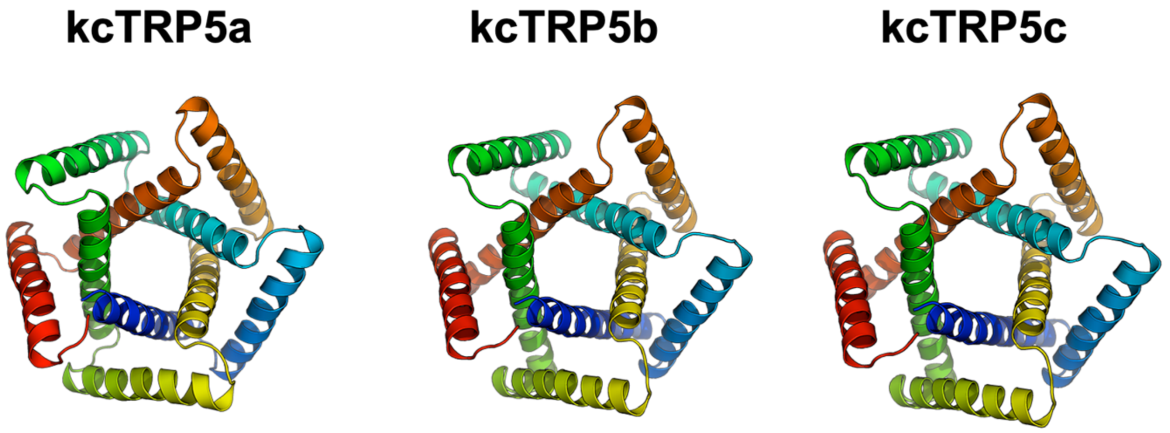

kcTRP5a

ATGGCTAGCAGCCATCATCATCATCATCATAGCAGCGGCCTGGTGCCGCGCGGCAGCTCCATGGGAAGGTCAATGGATGAACTAATAAAAAGACTC
GAGTATTTAGTTTTGGTGCTGCAACGCGAGGTTATTCTGGAGCAACAAGGTGAAAGCGAGGAACAGCTGCAGCGTAAGATCAGAAAGATCCAAGAG
AGCTTGAAGAAACAGGCGGAAGACGAAGGTCGCAGCCCGGATGAATTAATTAAACGTCTGCTGTACCTGGTTCTGGTCTTGCAGCGCGAGGTGATT
CTGGAACAGCAAGGCGAGTCCGAAGAACAGCTGCAACGTAAGATCCGCAAGATCCAGGAGTCTTTGAAAAAACAAGCTGAAGACGAGGGCCGCTC
GCCGGATGAGCTGATTAAACGTTTAAAATACCTGGTTCTGGTATTGCAGAGAGAAGAAATCCTGGAGCAGCAGGGCGAAAGCGAAGAGCAGCTGCA
GCGTAAGATCCGTAAAATCCAAGAGAGCCTTAAGAAACAGGCCGAAGATGAGGGTCGTTCCGAGGACGAGCTGGTTAAACGTTTGCTGTACTTGGT
GCTCGTCTTGCTGCGTGAAGTGATTCTGGAACAGCAGGGTGAGTCAGAGGAACAGCTGCAACGCAAGATTCGTAAGATTCAAGAAAGCCTGAAAAA
ACAAGCGGAGGACGAGGGCCGTAGCCCGGATGAGCTGACCAAACGCCTGCTGTATCTGGTGCTGGTGCTTGATCGTGAACGTATTTTGGAGCAAC
AGGGTGAGTCCGAGGAACAACTGCAGCGCAAAATCCGTAAGATCCAAGAATCTCTGAAGAAGCAGGCAGAGGACGAAGGCTAA

MASSHHHHHHSSGLVPRGSSMGRSMDELIKRLEYLVLVLQREVILEQQGESEEQLQRKIRKIQESLKKQAEDEGRSPDELIKRLLYLVLVLQREVILEQQGE
SEEQLQRKIRKIQESLKKQAEDEGRSPDELIKRLKYLVLVLQREEILEQQGESEEQLQRKIRKIQESLKKQAEDEGRSEDELVKRLLYLVLVLLREVILEQQGE
SEEQLQRKIRKIQESLKKQAEDEGRSPDELTKRLLYLVLVLDRERILEQQGESEEQLQRKIRKIQESLKKQAEDEG*

kcTRP5b

ATGGCTAGCAGCCATCATCATCATCATCATAGCAGCGGCCTGGTGCCGCGCGGCAGCTCCATGGGAGAATCAGAAGATGACTTAGAGAAACTACTG
CGGCTGCTGATGATTCGCTTGGAACTTCAGCGTCTGATCCGTGAATTGGCGAACGAGCAGGGTCTGAGCGAAGAGGAAGTGGCCCGTCGCCTGAA
CAAGGAACTGAAAGATGATACCCAACGTCTGCGTGAATCTGGCGAATCAGAAGATGACCTGCTCAAGCTGTTACAACTGTTATGGATTCGTTTGGAA
CTGCAGCGTCTTATCCGCGAGCTGGCTAACGAGCAGGGTTTGAGCGAGGAGGAGGTTGCGCGTCGTCTGAATAAAGAGCTAAAAGACGACACCCA
GCGCCTGCGCGAGAGCGGCGAATCCGAGGATGACCTGCTTAAGCTGCTGGCGCTGCTGCTCATCCGCTTATTGCTCCAGATTCTGATTAGAGAGCT
CGCTAATGAACAAGGTCTGTCGGAGGAGGAGGTGGCACGTCGTTTAAATAAGGAGTTGAAGGACGACACGCAGCGTCTGCGTGAATCTGGTGAAA
GCGAGGACGACCTGGAAAAACTGCTGCTGCTGCTTTGGATTCGCTTGGAGCTGCAACGTCTGATCCGTGAACTGGCAAACGAGCAAGGCCTGAGC
GAGGAGGAGGTTGCGCGTCGTCTGAATAAAGAACTTAAGGACGACACTCAACGCTTACGAGAGTCCGGCGAATCCGAAGATGATCTGACCAAACTG
CTGTTGCTGTTGTTCATCCGCCTGTTGCTTCAACAGTTGATCCGCGAACTCGCGAACGAACAGGGCCTGAGCGAGGAAGAGGTCGCCCGTCGTTTG
AACAAAGAACTGAAGGATGATACCCAGCGCCTGCGTGAAAGCGGTTAA

MASSHHHHHHSSGLVPRGSSMGESEDDLEKLLRLLMIRLELQRLIRELANEQGLSEEEVARRLNKELKDDTQRLRESGESEDDLLKLLQLLWIRLELQRLIR
ELANEQGLSEEEVARRLNKELKDDTQRLRESGESEDDLLKLLALLLIRLLLQILIRELANEQGLSEEEVARRLNKELKDDTQRLRESGESEDDLEKLLLLLWIR
LELQRLIRELANEQGLSEEEVARRLNKELKDDTQRLRESGESEDDLTKLLLLLFIRLLLQQLIRELANEQGLSEEEVARRLNKELKDDTQRLRESG*

kcTRP5c

ATGGCTAGCAGCCATCATCATCATCATCATAGCAGCGGCCTGGTGCCGCGCGGCAGCTCCATGGGAGAGTCAGAAGATGACCTATTAAAATTGCTG
ATGCTGCTGTGGATTCGCTTAATGCTGCAGATTCTGATTAGGGAGCTGGCTAATGAACAGGGTCTGTCTGAGGAAGAGGTGGCTCGTCGCCTCAAC
AAAGAACTCAAGGACGATACCCAGCGCCTCCGTGAGAGCGGCGAATCGGAGGATGATCTGCTTAAGCTGTTGCAACTGCTGTGGATTCGTCTGGAT
CTGCAGCGTCTGATCCGCGAGCTGGCAAATGAACAGGGCCTGAGCGAGGAAGAGGTCGCGCGTCGTTTAAACAAAGAATTGAAAGATGACACGCA
GCGTCTGCGTGAGTCCGGTGAAAGCGAAGACGACCTTGAGAAACTGCTGGCGCTGCTTTATATCAGATTGGAACTGCAACGCCTGATTCGCGAGCT
TGCGAACGAACAGGGTCTGAGCGAAGAAGAGGTTGCGCGTCGTTTGAATAAAGAACTCAAGGATGATACCCAACGCCTGCGTGAGTCCGGCGAAT
CCGAGGACGACCTGTTGAAGTTGTTGCTGTTGCTGTACATCCGTTTGGAGCTGCAACGTCTGATCCGTGAGTTAGCCAACGAGCAGGGTCTGAGCG
AGGAAGAAGTGGCACGTCGTCTGAACAAAGAGCTGAAGGACGACACTCAGCGTTTACGTGAATCTGGCGAAAGCGAGGATGATCTGCTTAAGCTGT
TGCTGCTGCTTTGGATCCGCCTCCAACTGCAACGACTGATCCGCGAGCTGAGCAATGAGCAAGGCCTGAGCGAGGAAGAAGTTGCGCGTCGTCTT
AACAAAGAACTGAAGGACGACACCCAGCGCCTGCGTGAGTCAGGTTAA

MASSHHHHHHSSGLVPRGSSMGESEDDLLKLLMLLWIRLMLQILIRELANEQGLSEEEVARRLNKELKDDTQRLRESGESEDDLLKLLQLLWIRLDLQRLIR
ELANEQGLSEEEVARRLNKELKDDTQRLRESGESEDDLEKLLALLYIRLELQRLIRELANEQGLSEEEVARRLNKELKDDTQRLRESGESEDDLLKLLLLLYI
RLELQRLIRELANEQGLSEEEVARRLNKELKDDTQRLRESGESEDDLLKLLLLLWIRLQLQRLIRELSNEQGLSEEEVARRLNKELKDDTQRLRESG*

Alignment of pentafoil designs

```
kcTRP5a      MASSHHHHHHSSGLVPRGSSMGRSMDELIKRLEYLVLVLQREVI----LEQQGESEEQLQ     56
kcTRP5b      MASSHHHHHHSSGLVPRGSSMGESEDDLEKLLRLLMIRLELQRLIRELANEQGLSEEEVA     60
kcTRP5c      MASSHHHHHHSSGLVPRGSSMGESEDDLLKLLMLLWIRLMLQILIRELANEQGLSEEEVA     60
             **********************.* *:* * *  * : *  : :     ::** ***::
                  (His-tag + thrombin site)

kcTRP5a      RKIRKIQESLKKQAEDEGRSPDELIKRLLYL--VLVLQRE--VILEQQGESEEQLQRKIR     112
kcTRP5b      RRLNKELKDDTQRLRESGESEDDLLKLLQLLWIRLELQRLIRELANEQGLSEEEVARRLN     120
kcTRP5c      RRLNKELKDDTQRLRESGESEDDLLKLLQLLWIRLDLQRLIRELANEQGLSEEEVARRLN     120
             *::.*  :. .:: .:.*.* *:*:* *  *   * ***    : ::** ***:: *::.

kcTRP5a      KIQESLKKQAEDEGRSPDELIKRLKYLVLVLVL----QREEILEQQGESEEQLQRKIRKIQE     168
kcTRP5b      KELKDDTQRLRESGESEDDLLKLLALLLIRLLLQILIRELANEQGLSEEEVARRLNKELK     180
kcTRP5c      KELKDDTQRLRESGESEDDLEKLLALLYIRLELQRLIRELANEQGLSEEEVARRLNKELK     180
             *  :. .:: .:.*.* *:* * *  * : *      .*: ::** ***:: *::.*  :

kcTRP5a      SLKKQAEDEGRSEDELVKRLLYLVLVLL----REVILEQQGESEEQLQRKIRKIQESLKK     224
kcTRP5b      DDTQRLRESGESEDDLEKLLLLLWIRLELQRLIRELANEQGLSEEEVARRLNKELKDDTQ     240
kcTRP5c      DDTQRLRESGESEDDLLKLLLLLYIRLELQRLIRELANEQGLSEEEVARRLNKELKDDTQ     240
             . .:: .:.*.***:* * ** * : *      . : ::** ***:: *::.*  :. .:

kcTRP5a      QAEDEGRSPDELTKRLLYLVLVLDRER----ILEQQGESEEQLQRKIRKIQESLKKQAED     280
kcTRP5b      RLRESGESEDDLTKLLLLLFIRLLLQQLIRELANEQGLSEEEVARRLNKELKDDTQRLRE     300
kcTRP5c      RLRESGESEDDLLKLLLLLWIRLQLQRLIRELSNEQGLSEEEVARRLNKELKDDTQRLRE     300
             : .:.*.* *:* * ** * : *  ::    : ::** ***:: *::.*  :. .:: .:

kcTRP5a      EG   282
kcTRP5b      SG   302
kcTRP5c      SG   302
              *
```

5. Second generation kcTRP5 ‘pentafoil’ designs

kcTRP5a.1

ATGGCTAGCAGCCATCATCATCATCATCATAGCAGCGGCCTGGTGCCGCGCGGCAGCTCCATGGGAGTATCACCCACTACACTAATAAC
GGAACGCGAGTACGTGGTTAATACCTTGGAAATCAAGAAGCTGAGAAAGGAGTTCGGCATTACGGAAGAGGAATTAAAGGAAGAAGAAAA
GCGCCTGCTGGAAGAGCTCAAGCAGACCTCGGAAGAGTTAGGTGTTAGCCCGACCACCCTGATTACCGAGCGCGAGTATGTTGTGAACA
CCCTGGAAATCAAAAAACTGCGTAAAGAATTCGGCATTACCGAAGAGGAGCTGAAGGAGGAGGAAAAGCGCCTGCTGGAAGAGCTGAAG
CAAACGTCCGAAGAGCTGGGTGTGAGCCCAACGACCCTGATCACCGAACGTGAATACGTCGTCAACACCTTGGAAATCAAAAAATTGCGT
AAAGAGTTTGGTATTACTGAAGAGGAGCTTAAGGAGGAAGAGAAACGTCTGCTGGAAGAGCTCAAGCAGACTAGCGAGGAACTTGGCGT
AAGCCCGACAACCCTGATTACTGAGCGCGAATACGTTGTTAACACCTTGGAGATCAAAAAACTGCGTAAAGAGTTCGGCATCACCGAAGA
GGAATTGAAAGAGGAGGAGAAACGTCTGCTGGAAGAACTGAAACAGACCTCTGAAGAGCTGGGCGTTAGCCCGACCACGCTTATCACGG
AACGTGAGTATGTGGTGAATACCTTGGAGATCAAGAAGCTGCGTAAAGAATTTGGTATTACCGAAGAGGAATTGAAGGAGGAGGAGAAGC
GTCTGTTGGAAGAACTGAAACAAACCTCCGAGGAGCTGGGTTGGTAA

MASSHHHHHHSSGLVPRGSSMGVSPTTLITEREYVVNTLEIKKLRKEFGITEEELKEEEKRLLEELKQTSEELGVSPTTLITEREYVVNTLEIKKLR
KEFGITEEELKEEEKRLLEELKQTSEELGVSPTTLITEREYVVNTLEIKKLRKEFGITEEELKEEEKRLLEELKQTSEELGVSPTTLITEREYVVNTLEI
KKLRKEFGITEEELKEEEKRLLEELKQTSEELGVSPTTLITEREYVVNTLEIKKLRKEFGITEEELKEEEKRLLEELKQTSEELGW*

kcTRP5a.2

ATGGCTAGCAGCCATCATCATCATCATCATAGCAGCGGCCTGGTGCCGCGCGGCAGCTCCATGGGAATAGATAAAAATACAATTATAAAG
AACTTGGAGTATGTGGTTAATACCTTGGAGATCGAACGCCTTCGCGAGGAGTTTGGCATCACCGAAGAGGAGCTGAAAGAGAAGGAAAA
GGCGATTCTGGCGGAATTAGACGAAACGTCCAAAAAGCTGGGTATTGATAAAAACACGATTATTAAAAACCTGGAGTATGTGGTCAACACC
TTGGAAATCGAGCGTCTGCGTGAAGAATTCGGCATCACTGAGGAGGAACTTAAGGAGAAGGAGAAGGCGATTCTGGCAGAACTCGACGA
GACCAGCAAAAAACTGGGCATCGATAAAAACACGATCATCAAGAATTTGGAGTACGTCGTGAATACCTTGGAGATCGAACGTCTGCGTGA
GGAATTTGGTATTACCGAAGAGGAACTGAAGGAGAAAGAGAAGGCTATCCTGGCGGAATTGGACGAGACAAGCAAGAAACTGGGCATTG
ATAAAAATACCATTATCAAGAACCTGGAATACGTTGTTAATACGTTAGAGATCGAACGTCTGAGAGAAGAGTTCGGCATCACCGAAGAAGA
GCTGAAAGAAAAAGAAAAGGCGATCTTGGCTGAACTGGATGAAACCAGCAAGAAACTGGGTATCGACAAGAACACCATTATTAAGAACCT
GGAATACGTTGTGAACACTCTCGAGATTGAGCGCCTGCGTGAAGAGTTCGGTATTACCGAAGAGGAGCTGAAAGAGAAAGAGAAGGCCA
TCCTGGCAGAACTGGACGAAACCTCTAAGAAATTGGGTTGGTAA

MASSHHHHHHSSGLVPRGSSMGIDKNTIIKNLEYVVNTLEIERLREEFGITEEELKEKEKAILAELDETSKKLGIDKNTIIKNLEYVVNTLEIERLREE
FGITEEELKEKEKAILAELDETSKKLGIDKNTIIKNLEYVVNTLEIERLREEFGITEEELKEKEKAILAELDETSKKLGIDKNTIIKNLEYVVNTLEIERLR
EEFGITEEELKEKEKAILAELDETSKKLGIDKNTIIKNLEYVVNTLEIERLREEFGITEEELKEKEKAILAELDETSKKLGW*

kcTRP5a.3

ATGGCTAGCAGCCATCATCATCATCATCATAGCAGCGGCCTGGTGCCGCGCGGCAGCTCCATGGGAGTATCAAGGGACACACTAATAAG
AGATCTAGAGTACGTTGTCGCGACCTTAGAGCTGGAACGCCTGCGTCGTGAATTCGGCATCTCCGAAGAAGAACTTGAGGCACGCGTGA
AAGCGCTGCTGGAGGAGTTGGAGCGCAGATCCGAGGAGCTGGGCGTTAGCCGTGACACCCTGATTAGAGATCTGGAGTATGTTGTTGCG
ACGCTGGAATTGGAGCGCCTGCGTCGTGAATTTGGCATCTCGGAAGAAGAGTTGGAGGCGCGTGTTAAGGCGCTGCTCGAAGAATTGGA
GCGCCGTAGCGAAGAGCTTGGCGTGAGCCGTGATACCCTGATCCGCGATCTGGAGTACGTCGTGGCCACGCTGGAGTTGGAACGCCTG
CGCCGTGAATTCGGTATCTCCGAAGAAGAATTGGAGGCACGTGTAAAAGCGCTGTTGGAAGAGCTGGAGCGACGTAGCGAGGAGCTGG
GTGTGTCTCGTGATACCCTGATTCGTGACTTGGAGTATGTGGTCGCTACCCTCGAGTTAGAACGTCTGCGTCGTGAATTTGGTATTAGCGA
AGAGGAATTGGAGGCACGTGTTAAAGCCTTGCTGGAAGAACTGGAGCGCCGTAGCGAAGAACTGGGTGTTAGCCGTGACACCCTTATCC
GTGACCTGGAATACGTTGTGGCGACTCTGGAACTCGAGCGCCTGCGTCGTGAGTTCGGCATTTCAGAAGAAGAGTTGGAGGCTCGCGTG
AAGGCTCTGCTGGAGGAATTAGAGCGTCGCTCTGAGGAGCTGGGTTGGTAA

MASSHHHHHHSSGLVPRGSSMGVSRDTLIRDLEYVVATLELERLRREFGISEEELEARVKALLEELERRSEELGVSRDTLIRDLEYVVATLELERL
RREFGISEEELEARVKALLEELERRSEELGVSRDTLIRDLEYVVATLELERLRREFGISEEELEARVKALLEELERRSEELGVSRDTLIRDLEYVVA
TLELERLRREFGISEEELEARVKALLEELERRSEELGVSRDTLIRDLEYVVATLELERLRREFGISEEELEARVKALLEELERRSEELGW*

kcTRP5b.1

ATGGCTAGCAGCCATCATCATCATCATCATAGCAGCGGCCTGGTGCCGCGCGGCAGCTCCATGGGAATATCAAGACGAGAGACACTAGA
AAGGCTATCGCTGCTGTTATTCTCCATGCAGCTGGAAAAGCTGGTTAAGGAGGAGGCTGAAGCGCGTGGTGTTAGCGTAGAGACTATCC
GCGAAGAGCTGGAGCGCGAGGTGGATGAGCGCCTGCGTGAGATGGAAGAGCAAGGCATTAGCCGCCGTGAAACCCTGGAGCGCTTGTC
TCTGCTGTTGTTTAGTATGCAGTTGGAAAAACTGGTCAAAGAAGAAGCAGAAGCGCGTGGCGTGAGCGTGGAAACCATTCGCGAGGAATT
GGAGCGTGAAGTTGACGAACGCCTGCGTGAAATGGAAGAGCAGGGCATCTCACGTCGTGAGACCCTGGAGCGTCTGAGCCTGCTGCTC
TTTTCCATGCAGCTGGAAAAGCTGGTGAAAGAGGAGGCCGAAGCACGTGGTGTTAGCGTCGAGACGATCCGTGAAGAGCTGGAGCGTGA
AGTGGACGAGCGTTTACGTGAGATGGAAGAGCAGGGTATCAGCAGACGTGAAACCCTTGAGCGCTTATCTCTGTTGTTGTTCTCCATGCA
ATTGGAGAAGCTGGTTAAAGAAGAGGCTGAAGCGCGTGGTGTGTCCGTGGAGACCATTCGTGAAGAGTTGGAGCGTGAGGTTGATGAAC
GCCTGCGCGAAATGGAAGAGCAAGGTATTAGCCGTCGTGAAACCTTGGAAAGACTGAGCCTGCTGCTGTTCTCTATGCAGTTGGAAAAGC
TGGTGAAAGAGGAGGCGGAAGCGCGTGGCGTCAGCGTTGAAACGATCCGCGAGGAACTCGAGCGTGAAGTTGACGAGCGCCTGCGTGA
GATGGAAGAACAAGGTTGGTAA

MASSHHHHHHSSGLVPRGSSMGISRRETLERLSLLLFSMQLEKLVKEEAEARGVSVETIREELEREVDERLREMEEQGISRRETLERLSLLLFSM
QLEKLVKEEAEARGVSVETIREELEREVDERLREMEEQGISRRETLERLSLLLFSMQLEKLVKEEAEARGVSVETIREELEREVDERLREMEEQG
ISRRETLERLSLLLFSMQLEKLVKEEAEARGVSVETIREELEREVDERLREMEEQGISRRETLERLSLLLFSMQLEKLVKEEAEARGVSVETIREEL
EREVDERLREMEEQGW*

kcTRP5b.2

ATGGCTAGCAGCCATCATCATCATCATCATAGCAGCGGCCTGGTGCCGCGCGGCAGCTCCATGGGAGTATCAAAGAAAGAATATCTAGA
GAGGTTGGCGCTGCTGCTGTATACCCTGGAGTTGGAAAAGCTCATTCGTGAAAAGGCTGAAGAGCTGGGCGTATCTGTGGAAGAGATCC
GCGAGCGTCTGGAGAAAGAAGTGGAAGAGCGTGAGGAGGAAATGAAGAAGAAGGGCGTCAGCAAAAAAGAATATCTGGAACGTCTGGC
ACTGTTACTGTACACGCTGGAGCTGGAAAAGCTGATCCGTGAAAAGGCCGAAGAACTCGGCGTGAGCGTTGAGGAGATTCGCGAGAGAT
TGGAGAAAGAAGTTGAAGAGCGCGAAGAGGAGATGAAAAAGAAGGGTGTGTCGAAAAAAGAGTACTTGGAGCGCCTGGCTCTTCTGCTG
TATACCCTGGAACTTGAGAAGCTGATTCGTGAGAAGGCGGAAGAATTAGGCGTGAGCGTTGAAGAAATCCGTGAACGCCTGGAAAAGGA
GGTTGAAGAGCGCGAAGAGGAGATGAAAAAGAAAGGCGTCAGCAAAAAAGAATATCTGGAACGTTTGGCGCTGTTGTTGTACACCCTGG
AACTCGAGAAACTGATTCGTGAGAAGGCGGAAGAGCTGGGTGTCTCCGTGGAGGAAATCCGCGAACGTCTGGAGAAAGAAGTTGAGGAG
CGTGAGGAAGAAATGAAAAAGAAAGGTGTGTCCAAAAAAGAATACCTGGAGAGACTGGCGCTGCTGTTGTACACTTTGGAGTTGGAGAAA
CTGATCCGTGAAAAGGCAGAAGAGTTAGGTGTTAGCGTTGAAGAGATCCGTGAACGTTTGGAGAAGGAGGTGGAAGAGCGCGAGGAAGA
GATGAAAAAGAAGGGTTGGTAA

MASSHHHHHHSSGLVPRGSSMGVSKKEYLERLALLLYTLELEKLIREKAEELGVSVEEIRERLEKEVEEREEEMKKKGVSKKEYLERLALLLYTL
ELEKLIREKAEELGVSVEEIRERLEKEVEEREEEMKKKGVSKKEYLERLALLLYTLELEKLIREKAEELGVSVEEIRERLEKEVEEREEEMKKKGVS
KKEYLERLALLLYTLELEKLIREKAEELGVSVEEIRERLEKEVEEREEEMKKKGVSKKEYLERLALLLYTLELEKLIREKAEELGVSVEEIRERLEKE
VEEREEEMKKKGW*

kcTRP5b.3

ATGGCTAGCAGCCATCATCATCATCATCATAGCAGCGGCCTGGTGCCGCGCGGCAGCTCCATGGGAGAATCAAGAGAAGAATATCTAGA
GAGGCTCGAGTACTTGCTGGGTAAACTGCAACTGGAGGAGTTAATTAAAAAGAAGGCAGAAGAATTAGGCCTGAGCGTTGAAACGGTTCG
TAAAGAACTTGAGAAAGAAGTTGAGGAAGAGGAAAAACGTATGGAGGAGGCCGGTGAAAGCCGTGAAGAGTACTTGGAACGTCTGGAGT
ATCTGCTGGGTAAGCTGCAATTGGAGGAGTTGATTAAGAAGAAGGCGGAAGAGCTGGGCCTGTCCGTTGAAACCGTGCGTAAAGAATTG
GAGAAAGAGGTCGAAGAGGAGGAGAAGCGCATGGAAGAAGCTGGCGAGTCGAGAGAAGAATACCTCGAGCGCCTGGAATATCTGTTGG
GTAAACTGCAGCTGGAGGAGTTGATCAAAAAGAAAGCGGAAGAACTTGGCCTGTCTGTCGAAACTGTACGCAAAGAATTGGAGAAGGAAG
TGGAAGAGGAGGAGAAACGTATGGAAGAGGCGGGTGAAAGCCGTGAGGAGTACCTGGAGCGCCTGGAATATCTGCTGGGCAAACTGCA
GCTGGAAGAGCTTATCAAGAAAAAGGCTGAAGAGTTGGGCCTCAGCGTTGAAACCGTGCGCAAGGAGTTGGAGAAGGAAGTTGAAGAGG
AGGAGAAGCGTATGGAAGAAGCGGGTGAAAGCCGTGAAGAGTACCTGGAACGTCTGGAGTATTTGCTGGGCAAACTGCAGCTGGAAGA
GCTGATCAAAAAAAAGGCGGAAGAGCTGGGTCTGTCCGTGGAAACCGTGCGTAAAGAACTGGAGAAGGAGGTGGAAGAGGAGGAAAAG
CGCATGGAAGAGGCAGGTTGGTAA

MASSHHHHHHSSGLVPRGSSMGESREEYLERLEYLLGKLQLEELIKKKAEELGLSVETVRKELEKEVEEEEKRMEEAGESREEYLERLEYLLGK
LQLEELIKKKAEELGLSVETVRKELEKEVEEEEKRMEEAGESREEYLERLEYLLGKLQLEELIKKKAEELGLSVETVRKELEKEVEEEEKRMEEAG

ESREEYLERLEYLLGKLQLEELIKKKAEELGLSVETVRKELEKEVEEEEKRMEEAGESREEYLERLEYLLGKLQLEELIKKKAEELGLSVETVRKEL
EKEVEEEEKRMEEAGW*

Alignment of second generation pentafoil designs

```
kcTRP5a      MASSHHHHHHSSGLVPRGSSMGRSMDELIKRLEYLVLVLQREVILEQQGESEEQLQRKIR  60
kcTRP5a.1    MASSHHHHHHSSGLVPRGSSMGVSPTTLITEREYVVNTLEIKKLRKEFGITEEELKEEEK  60
kcTRP5a.2    MASSHHHHHHSSGLVPRGSSMGIDKNTIIKNLEYVVNTLEIERLREEFGITEEELKEKEK  60
kcTRP5a.3    MASSHHHHHHSSGLVPRGSSMGVSRDTLIRDLEYVVATLELERLRREFGISEEELEARVK  60
             ********************** .   :*   **:* .*: : : .: * :**:*: . :
               (His-tag + thrombin site)

kcTRP5a      KIQESLKKQAEDEGRSPDELIKRLLYLVLVLQREVILEQQGESEEQLQRKIRKIQESLKK  120
kcTRP5a.1    RLLEELKQTSEELGVSPTTLITEREYVVNTLEIKKLRKEFGITEEELKEEEKRLLEELKQ  120
kcTRP5a.2    AILAELDETSKKLGIDKNTIIKNLEYVVNTLEIERLREEFGITEEELKEKEKAILAELDE  120
kcTRP5a.3    ALLEELERRSEELGVSRDTLIRDLEYVVATLELERLRREFGISEEELEARVKALLEELER  120
              :  .*.. ::. * .   :*    *:* .*: : : .: * :**:*: . : :  .*..

kcTRP5a      QAEDEGRSPDELIKRLKYLVLVLQREEILEQQGESEEQLQRKIRKIQESLKKQAEDEGRS  180
kcTRP5a.1    TSEELGVSPTTLITEREYVVNTLEIKKLRKEFGITEEELKEEEKRLLEELKQTSEELGVS  180
kcTRP5a.2    TSKKLGIDKNTIIKNLEYVVNTLEIERLREEFGITEEELKEKEKAILAELDETSKKLGID  180
kcTRP5a.3    RSEELGVSRDTLIRDLEYVVATLELERLRREFGISEEELEARVKALLEELERRSEELGVS  180
              ::. * .   :*   :*:* .*: :.: .: * :**:*: . : :  .*.. ::. * .

kcTRP5a      EDELVKRLLYLVLVLLREVILEQQGESEEQLQRKIRKIQESLKKQAEDEGRSPDELTKRL  240
kcTRP5a.1    PTTLITEREYVVNTLEIKKLRKEFGITEEELKEEEKRLLEELKQTSEELGVSPTTLITER  240
kcTRP5a.2    KNTIIKNLEYVVNTLEIERLREEFGITEEELKEKEKAILAELDETSKKLGIDKNTIIKNL  240
kcTRP5a.3    RDTLIRDLEYVVATLELERLRREFGISEEELEARVKALLEELERRSEELGVSRDTLIRDL  240
                ::    *:* .*  : : .: * :**:*: . : :  .*.. ::. * .   :

kcTRP5a      LYLVLVLDRERILEQQGESEEQLQRKIRKIQESLKKQAEDEG*-   282
kcTRP5a.1    EYVVNTLEIKKLRKEFGITEEELKEEEKRLLEELKQTSEELGW*   283
kcTRP5a.2    EYVVNTLEIERLREEFGITEEELKEKEKAILAELDETSKKLGW*   283
kcTRP5a.3    EYVVATLELERLRREFGISEEELEARVKALLEELERRSEELGW*   283
              *:* .*: ::: .: * :**:*: . : :  .*.. ::. *


kcTRP5b      MASSHHHHHHSSGLVPRGSSMGESEDDLEKLLRLLMIRLELQRLIRELANEQGLSEEEVA  60
kcTRP5b.1    MASSHHHHHHSSGLVPRGSSMGISRRETLERLSLLLFSMQLEKLVKEEAEARGVSVETIR  60
kcTRP5b.2    MASSHHHHHHSSGLVPRGSSMGVSKKEYLERLALLLYTLELEKLIREKAEELGVSVEEIR  60
kcTRP5b.3    MASSHHHHHHSSGLVPRGSSMGESREEYLERLEYLLGKLQLEELIKKKAEELGLSVETVR  60
             ********************** *. :  : *  *:  ::*:.*::: *:  *:* * :
               (His-tag + thrombin site)

kcTRP5b      RRLNKELKDDTQRLRESGESEDDLLKLLQLLWIRLELQRLIRELANEQGLSEEEVARRLN  120
kcTRP5b.1    EELEREVDERLREMEEQGISRRETLERLSLLLFSMQLEKLVKEEAEARGVSVETIREELE  120
kcTRP5b.2    ERLEKEVEEREEEMKKKGVSKKEYLERLALLLYTLELEKLIREKAEELGVSVEEIRERLE  120
kcTRP5b.3    KELEKEVEEEEKRMEEAGESREEYLERLEYLLGKLQLEELIKKKAEELGLSVETVRKELE  120
             ..*::*:.:  ..:.: * *. : *: *  *   ::*:.*::: *:  *:* * : ..*:

kcTRP5b      KELKDDTQRLRESGESEDDLLKLLALLLIRLLLQILIRELANEQGLSEEEVARRLNKELK  180
kcTRP5b.1    REVDERLREMEEQGISRRETLERLSLLLFSMQLEKLVKEEAEARGVSVETIREELEREVD  180
kcTRP5b.2    KEVEEREEEMKKKGVSKKEYLERLALLLYTLELEKLIREKAEELGVSVEEIRERLEKEVE  180
kcTRP5b.3    KEVEEEEKRMEEAGESREEYLERLEYLLGKLQLEELIKKKAEELGLSVETVRKELEKEVE  180
             :*:.:  ..:.: * *. : *: *  **  : *: *::: *:  *:* * : ..*::*:.

kcTRP5b      DDTQRLRESGESEDDLEKLLLLLWIRLELQRLIRELANEQGLSEEEVARRLNKELKDDTQ  240
kcTRP5b.1    ERLREMEEQGISRRETLERLSLLLFSMQLEKLVKEEAEARGVSVETIREELEREVDERLR  240
kcTRP5b.2    EREEEMKKKGVSKKEYLERLALLLYTLELEKLIREKAEELGVSVEEIRERLEKEVEEREE  240
kcTRP5b.3    EEEKRMEEAGESREEYLERLEYLLGKLQLEELIKKKAEELGLSVETVRKELEKEVEEEEK  240
             :  ..:.: * *. :  : *  *   ::*:.*::: *:  *:* * : ..*::*:.:  .

kcTRP5b      RLRESGESEDDLTKLLLLLFIRLLLQQLIRELANEQGLSEEEVARRLNKELKDDTQRLRE  300
kcTRP5b.1    EMEEQGISRRETLERLSLLLFSMQLEKLVKEEAEARGVSVETIREELEREVDERLREMEE  300
kcTRP5b.2    EMKKKGVSKKEYLERLALLLYTLELEKLIREKAEELGVSVEEIRERLEKEVEEREEEMKK  300
```

```
kcTRP5b.3     RMEEAGESREEYLERLEYLLGKLQLEELIKKKAEELGLSVETVRKELEKEVEEEEKRMEE  300
              .:.: * *. :  : *  *:  : *::*::: *:  *:* * : ..*::*:.:  ..:.:

kcTRP5b       SG*-    302
kcTRP5b.1     QGW*    303
kcTRP5b.2     KGW*    303
kcTRP5b.3     AGW*    303
               *
```

Supplementary Figures

Supplementary Figure 1. Protein expression constructs. ***Panel a:*** Open reading frame encoding kcTRP3d (above) and plasmid map (below). kcTRP3a-c differ only in design sequence starting at codon 24. Repeating sequences are boxed. The numbers to the right correspond to the base pair and amino acid number. Plasmid map made using SnapGene software (from Insightful Science; available at snapgene.com) ***Panel b:*** Cloning region sequence for pET15HE. Stock vector contains a 19 base pair 'Stuffer' sequence between the *Nco*I and *Not*I cloning sites to allow for quick purification of linearized vector using kits designed for PCR reaction clean-up.

a

```
ATG GCT AGC AGC CAT CAT CAT CAT CAT CAT AGC AGC GGC CTG GTG CCG CGC GGC AGC TCC  60
M   A   S   S   H   H   H   H   H   H   S   S   G   L   V   P   R   G   S   S    20

ATG GGT AGC GAC GAA CAG CGT CGT GAG CTG GAG GAA AAA ATC AAG AAA AAG CTG GAG GAA  120
M   G   S   D   E   Q   R   R   E   L   E   E   K   I   K   K   K   L   E   E    40

CTG AAA ACC AAG AGC GAG GAA GAG CGT AAA GAA ATC AAG CTG CGT GTG ATT GCG TAC GTG  180
L   K   T   K   S   E   E   E   R   K   E   I   K   L   R   V   I   A   Y   V    60

CTG GTT CAG CTG GAG GAC CTG CAG AAG AAC CTG AGC GAT GAA CAA CGT CGT GAG CTG GAA  240
L   V   Q   L   E   D   L   Q   K   N   L   S   D   E   Q   R   R   E   L   E    80

GAG AAG ATT AAA AAG AAA CTG GAA GAG CTG AAA ACC AAG AGC GAA GAG GAA CGC AAG GAA  300
E   K   I   K   K   K   L   E   E   L   K   T   K   S   E   E   E   R   K   E    100

ATC AAG CTG CGT GTT ATT GCG TAT GTT CTG GTG CAA CTG GAA GAC CTG CAG AAG AAC TTA  360
I   K   L   R   V   I   A   Y   V   L   V   Q   L   E   D   L   Q   K   N   L    120

AGC GAT GAG CAA CGT CGT GAA CTG GAA GAA AAG ATC AAG AAA AAG CTG GAA GAA CTG AAG  420
S   D   E   Q   R   R   E   L   E   E   K   I   K   K   K   L   E   E   L   K    140

ACC AAA AGC GAG GAA GAG CGC AAG GAG ATT AAA CTG CGC GTT ATC GCG TAT GTT CTG GTT  480
T   K   S   E   E   E   R   K   E   I   K   L   R   V   I   A   Y   V   L   V    160

CAG CTG GAG GAT CTG CAA AAG AAC CTG AGC TAA  513
Q   L   E   D   L   Q   K   N   L   S   *    171
```

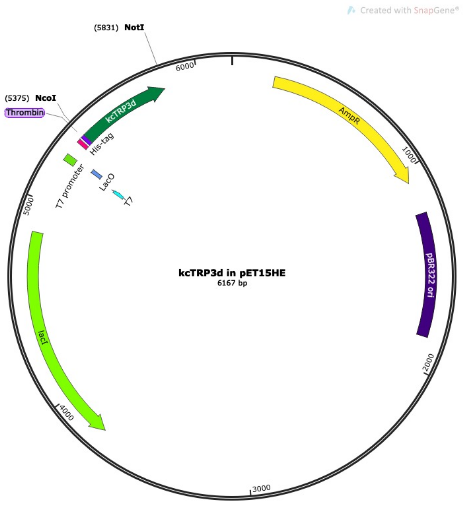

b

```
                T7 promoter            lac operator                                    RBS
5225 TAATACGACTCACTATAGGGGAATTGTGAGCGGATAACAATTCCCCTCTAGAAATAATTTTGTTTAACTTTAAGAAGGAGATATACT

start       His Tag                   Thrombin          NcoI              NotI   stop
ATGGCTAGCAGCCATCATCATCATCATCATAGCAGCGGCCTGGTGCCGCGCGGCAGCTCCATGGAGTTAATTAAGAGCGGCCGCTAG 5398
MetAlaSerSerHisHisHisHisHisHisSerSerGlyLeuValProArgGlySerSerMet... Stuffer
```

Supplementary Figure 2. Generation and behavior of first-generation knotted trefoil designs (kcTRP3a to 3d). ***Panel a***: Protein expression. (-) indicates pre-induction samples; (+) indicates post-induction samples. Arrows indicated position of visibly expressed protein or, in the case of kcTRP3a, approximate location expected. ***Panel b***: His-tag metal affinity purification. L = lysate, CL = cleared lysate, FT = column flow-through. ***Panel c:*** Size exclusion chromatographic purification of kcTRP3c (purple) and kcTRP3d (orange). Size exclusion standards are indicated by gray dotted lines. Inset illustrates final purified product purity. Source data are provided as a Source Data file.

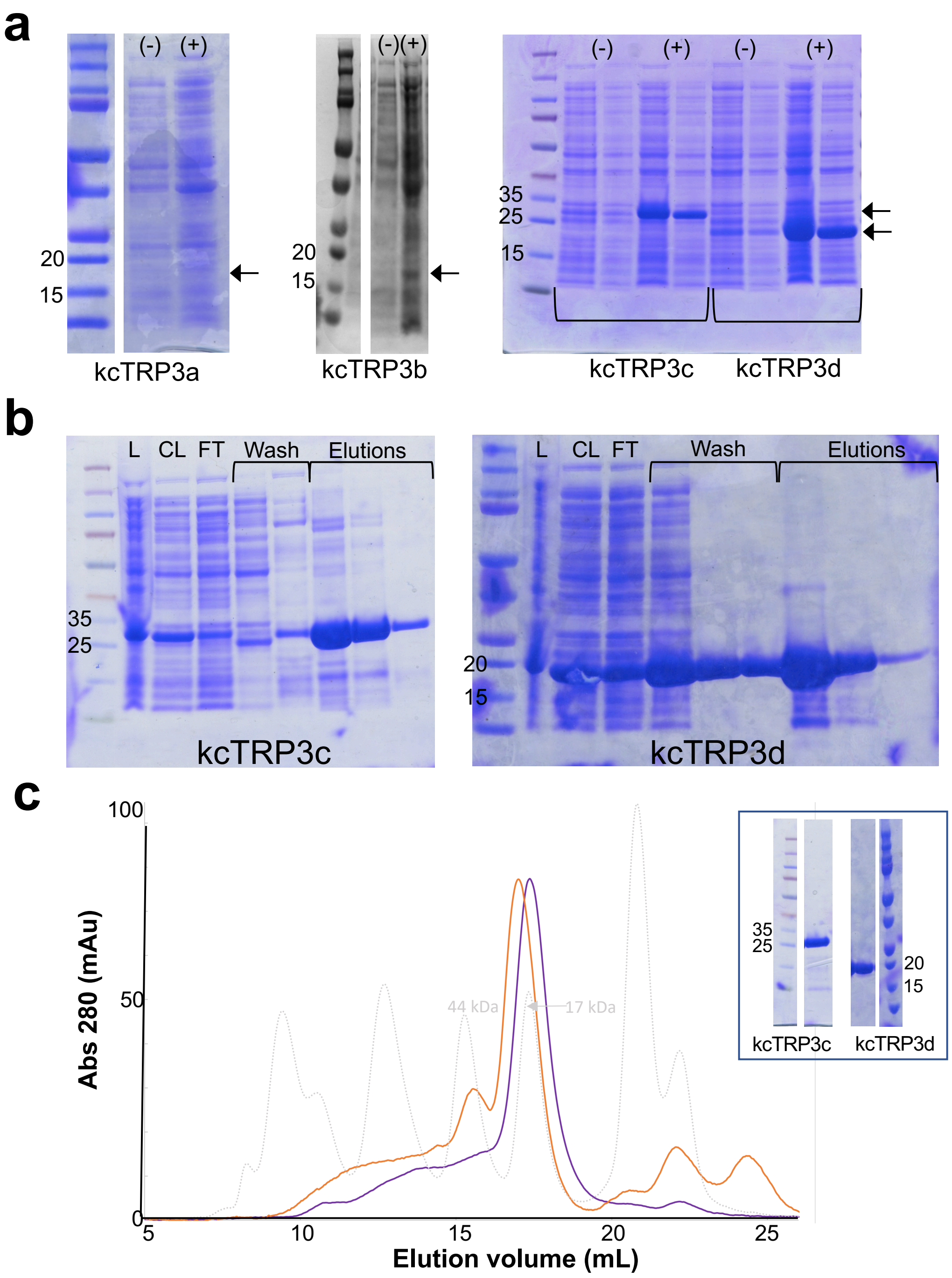

Supplementary Figure 3. Circular dichroism spectra of kcTRP3c (***panel a, top***) and kcTRP3d (***panel b, bottom***). Spectra were collected at 22° C (blue) and 95° C (red) and indicate significant retention of secondary structure at 95°. Source data are provided as a Source Data file.

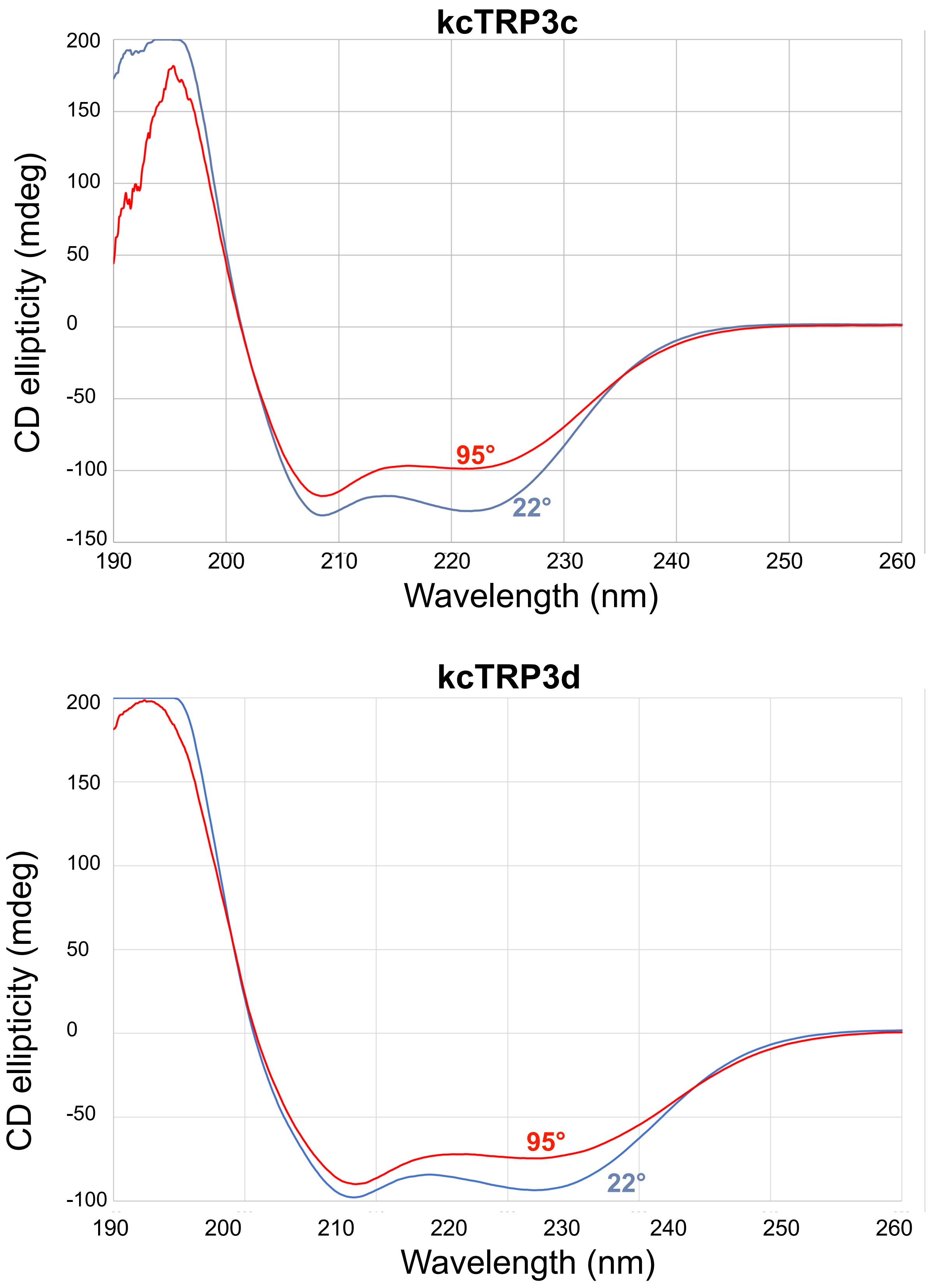

Supplementary Figure 4. **Size exclusion chromatographic behavior of second-generation trefoils** (kcTRP3d.1, kcTRP3d.2 and kcTRP3d.3) used for subsequent crystallographic structure determinations (**Figure 2**). Traces coorespond to kcTRP3d.1 (yellow), kcTRP3d.2 (brown), and kcTRP3d.3 (orange), with SEC Standards in dotted gray. Gel inset shows concentrated, post-SEC proteins. Source data are provided as a Source Data file.

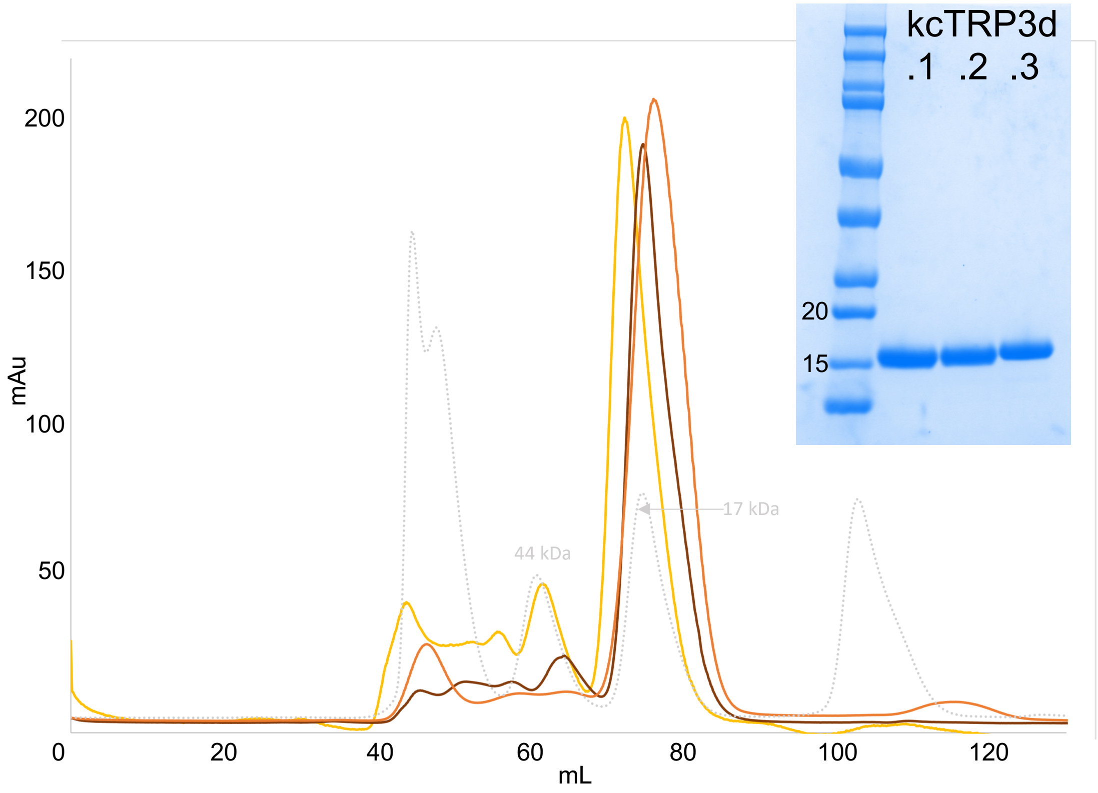

Supplementary Figure 5. Further characterization of the GdmCl-induced unfolding and refolding of designed knotted proteins: equilibrium and kinetic experiments. ***Panel a:*** Average emission wavelength for kcTRP3d.2 at pH 7.5, 25 ºC measured after 24 h (solid blue circles) and 48 h (solid red circles) in GdmCl. ***Panel b:*** Average emission wavelength for kcTRP3d.3 at pH 7.5, 25 ºC measured after 24 h (solid blue circles) and 72 h (solid red circles) in GdmCl. Data were fitted to a three-state unfolding model and the best fit is shown (dashed lines). To facilitate the fitting to a three-state model, the AEW value of the highest concentration was assumed to be the same at a higher concentration (9 M), although this was not measured. ***Panel c:*** Repeated measurements of the average emission wavelength for kcTRP3d.3 at pH 7.5 at 25 ºC measured after 24 h (solid red circles and solid blue circles), and at 10 ºC (solid green circles). All datasets were fit to a three-state unfolding model and the best fits are shown by the solid black lines. ***Panel d:*** The average emission wavelength (solid blue circles) or ellipticity at 222 nm (solid red circles) of kcTRP3d.2 as a function of GdmCl concentration. The dashed black lines show the best fit of the fluorescence and far-UV CD data to a three- and two-state unfolding model, respectively. To facilitate the fitting to a three-state model, the AEW value of the highest concentration was assumed to be the same at a higher concentration (9 M), although this was not measured. ***Panel e:*** The ellipticity at 222 nm (solid red circles) of kcTRP3d.1 as a function of GdmCl concentration. The solid black line shows the best fit of the far-UV CD data to a two-state unfolding model. For kcTRP3d.1, fluorescence data was not measured as there are only three tyrosines and no tryptophan residues in this construct so the change in fluorescence on unfolding was too small for accurate stability measurements to be made. ***Panel f:*** The average emission wavelength (solid blue circles) or ellipticity at 222 nm (solid red circles) of kcTRP3d.3 at pH 4.0 are shown as a function of GdmCl concentration. The solid black lines show the best fit of the fluorescence and far-UV CD data to a three-state unfolding model. ***Panels g and h:*** Refolding rate constants of kcTRP3d.3 as a function of protein concentration. Refolding kinetics were measured at a final concentration of 1.2 M GdmCl, pH 7.5, 25 ºC and data fitted to a double exponential to obtain rate constants (***panel g***) and amplitudes (***panel h***) for the two phases observed. The rate constants and amplitudes for the faster of the two kinetic refolding phases (k_1 and A_1, respectively), are shown as solid red circles, whilst the rate constants and amplitudes of the slightly slower refolding phase (k_2 and A_2, respectively), are shown as solid blue circles. The error bars show the standard deviations from multiple runs. ***Panels i and j:*** Refolding kinetics of kcTRP3d.3 from the intermediate state populated under equilibrium conditions at pH 7.5, 25 ºC. kcTRP3d.3 was initially denatured in 6.2 M GdmCl. In this case, a double exponential analysis was used to analyse all the data between final GdmCl concentrations of 1 to 4.5 M. ***Panel i***: The rate constants for the faster of the two kinetic refolding phases, called k_1, are shown as solid red circles, whilst the rate constants of the slightly slower refolding phase, termed k_2, are shown as solid blue circles. The error bars show the standard deviations from multiple runs. ***Panel j:*** Relative amplitudes of the two refolding phases shown as a percentage of the overall change in signal on refolding. Error bars are standard deviations calculated from multiple measurements under the same conditions. Symbols are as used in ***panel i***. Source data are provided as a Source Data file.

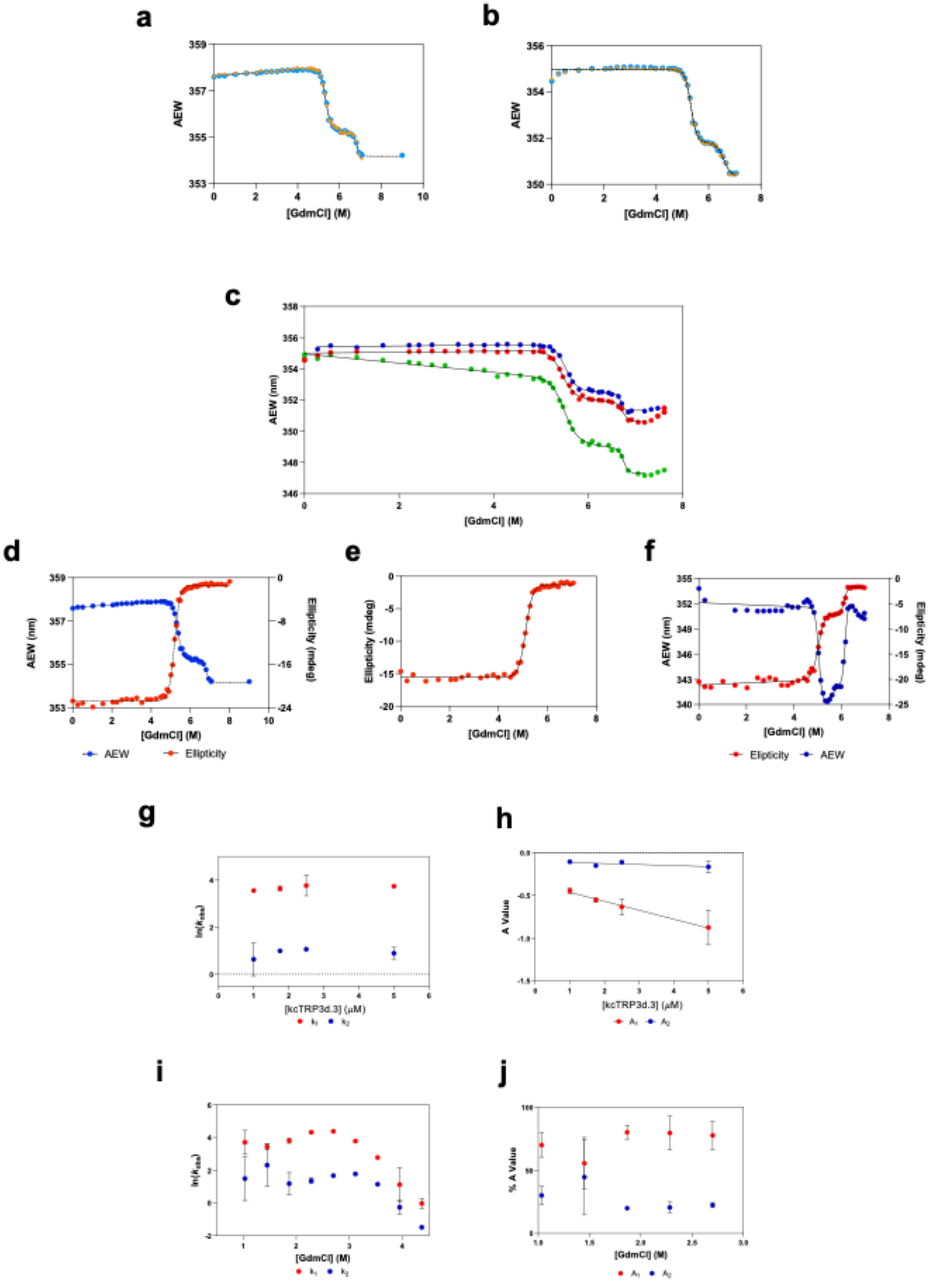
a
b
c
d
e
f
g
h
i
j
AEW
AEW (nm)
[GdmCl] (M)
Ellipticity (mdeg)
AEW
Ellipticity
Elipticity
ln(k_{obs})
[kcTRP3d.3] (μM)
k_1
k_2
A Value
A_1
A_2
% A Value

Supplementary Figure 6. Generation and behavior of knotted pentafoil designs (kcTRP5a to 5c). ***Panel a***: Protein expression. (-) indicates pre-induction samples; (+) indicates post-induction samples. Arrows indicated position of visibly expressed protein. ***Panel b***: Strep-tag (kcTRP5a and 5b) or His-tag (kcTRP5c) affinity purification. L = lysate, CL = cleared lysate, FT = column flow-through. ***Panel c:*** Size exclusion chromatographic purification of kcTRP5a (purple) and kcTRP5b (orange). Size exclusion standards are indicated by gray dotted lines. Inset illustrates final purified product purity. Source data are provided as a Source Data file.

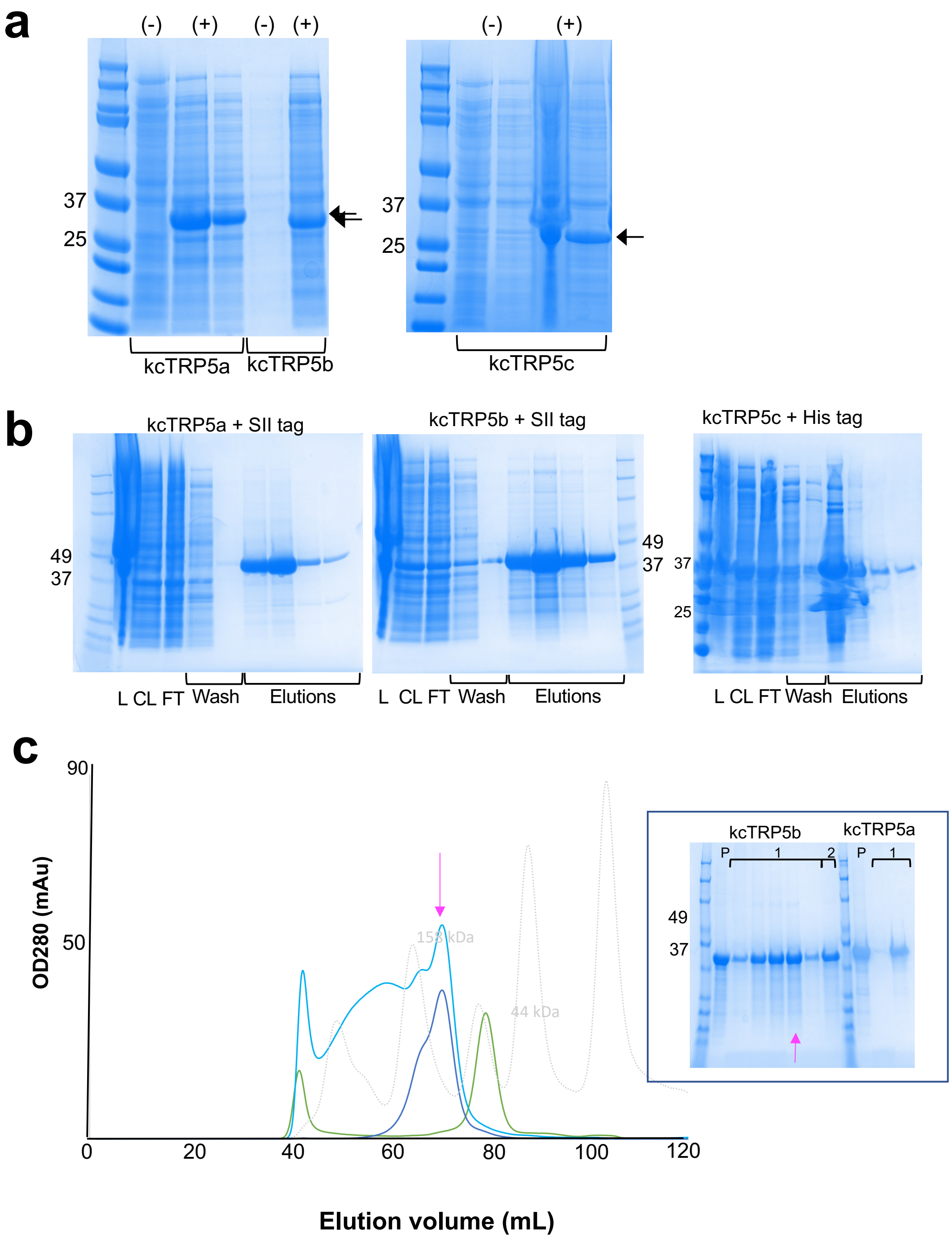

Supplementary Figure 7. SAXS analysis of solution behavior of designed pentafoil kcTRP5a. ***Panel a:*** The theoretical SAXS profile of the design model (red line) was fit to the experimental data (black circles). The scattering intensity is plotted on a log scale against the scattering vector q and the standard deviation of the measurements at each q is plotted as gray shading. The quality of this fit (chi^2) was 0.12 and the theoretical scattering profile is contained within the error. The residual of the model to the experimental data (bottom) is plotted on a linear scale to better represent the magnitude of the deviation across the q range. ***Panel b:*** Dimensionless Kratky plot of experimental SAXS data (black circles) overlayed with the theoretical spectrum calculated from the design model (red line). The raised and plateaued experimental at q*Rg > 3.5 indicates flexibility of the protein in the sample in relation to the perfectly rigid design model. Rg is radius of gyration. I(0) is the extrapolated initial scattering intensity.

a

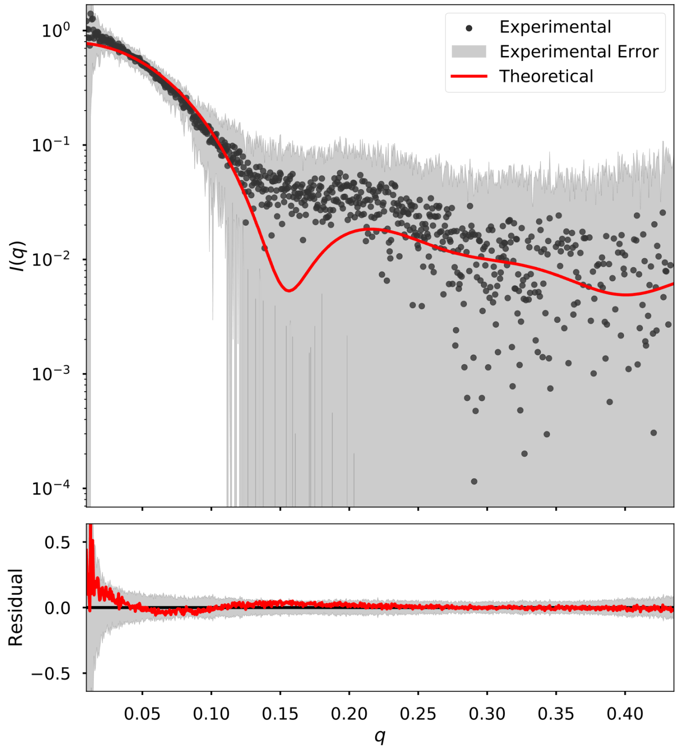

b

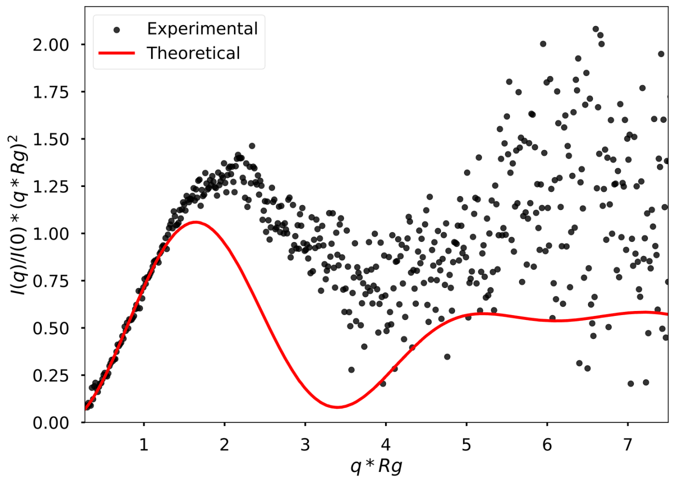

Supplementary Figure 8. Comparison of original designs of trefoil and pentafoil constructs with subsequent prediction of their topologies from the designed sequences using the AlphaFold structure prediction server. Whereas the topology of three of the four trefoil designs is predicted by the algorithm, all three pentafoils are instead predicted to form unknotted protein folds.

kcTRP3a 1.7Å C_{α} RMSD between design model (L) and alphafold prediction (R)

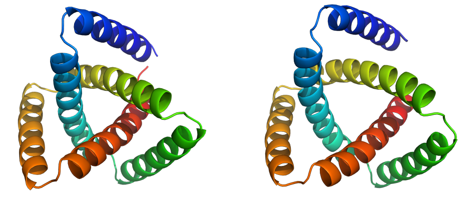

kcTRP3b 15.8Å C_{α} RMSD between design model (L) and alphafold prediction (R)

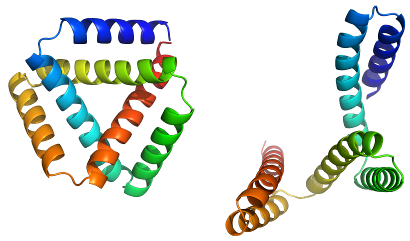

kcTRP3c 2.9Å C_{α} RMSD between design model (L) and alphafold prediction (R)

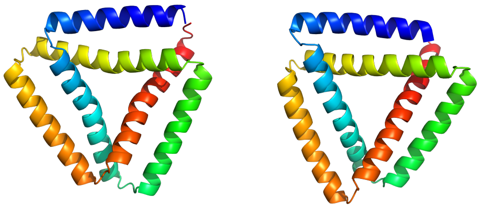

kcTRP3d 1.2Å C_{α} RMSD between design model (L) and alphafold prediction (R)

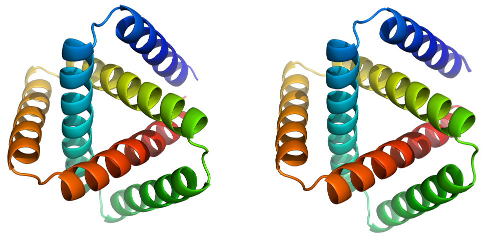

kcTRP5a 22.9Å C_{α} RMSD between design model (L) and alphafold prediction (R)

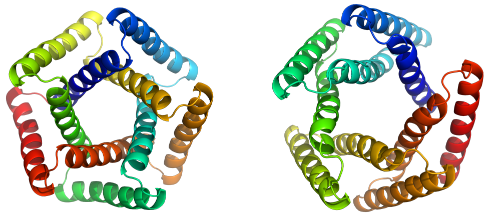

kcTRP5b 24.3Å C_{α} RMSD between design model (L) and alphafold prediction (R)

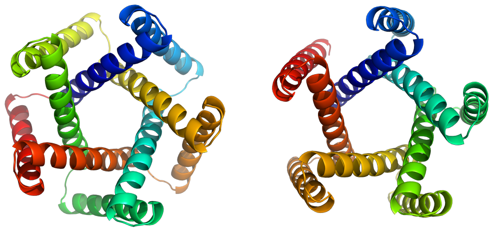

kcTRP5c 24.3Å C_{α} RMSD between design model (L) and alphafold prediction (R)

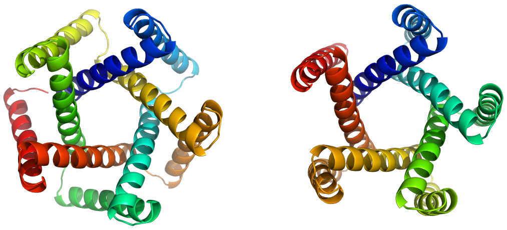

Supplementary Figure 9. Generation and behavior of second generation knotted pentafoil designs (kcTRP5a.2, kcTRP5a.3 and kcTRP5b.1). ***Panel a***: Expression of second generation kcTRP5. All lanes are post-induction. Black arrows highlight expressed protein. ***Panel b***: Visualization of affinity purification. L = lysate, CL = cleared lysate, FT = column flow-through. ***Panel c***: Size exclusion chromatography of kcTRP5a.2 (yellow) with peak collected (yellow arrow), kcTRP5a.3 (magenta) with trailing edge of peak collected (magenta arrow), and kcTRP5b.1 (blue) with peak collected (blue arrow). Size exclusion standards are gray dotted. Inset gel of peak fractions. P = pre-column sample. Arrow indicates peak fraction collected for crystallization trials.

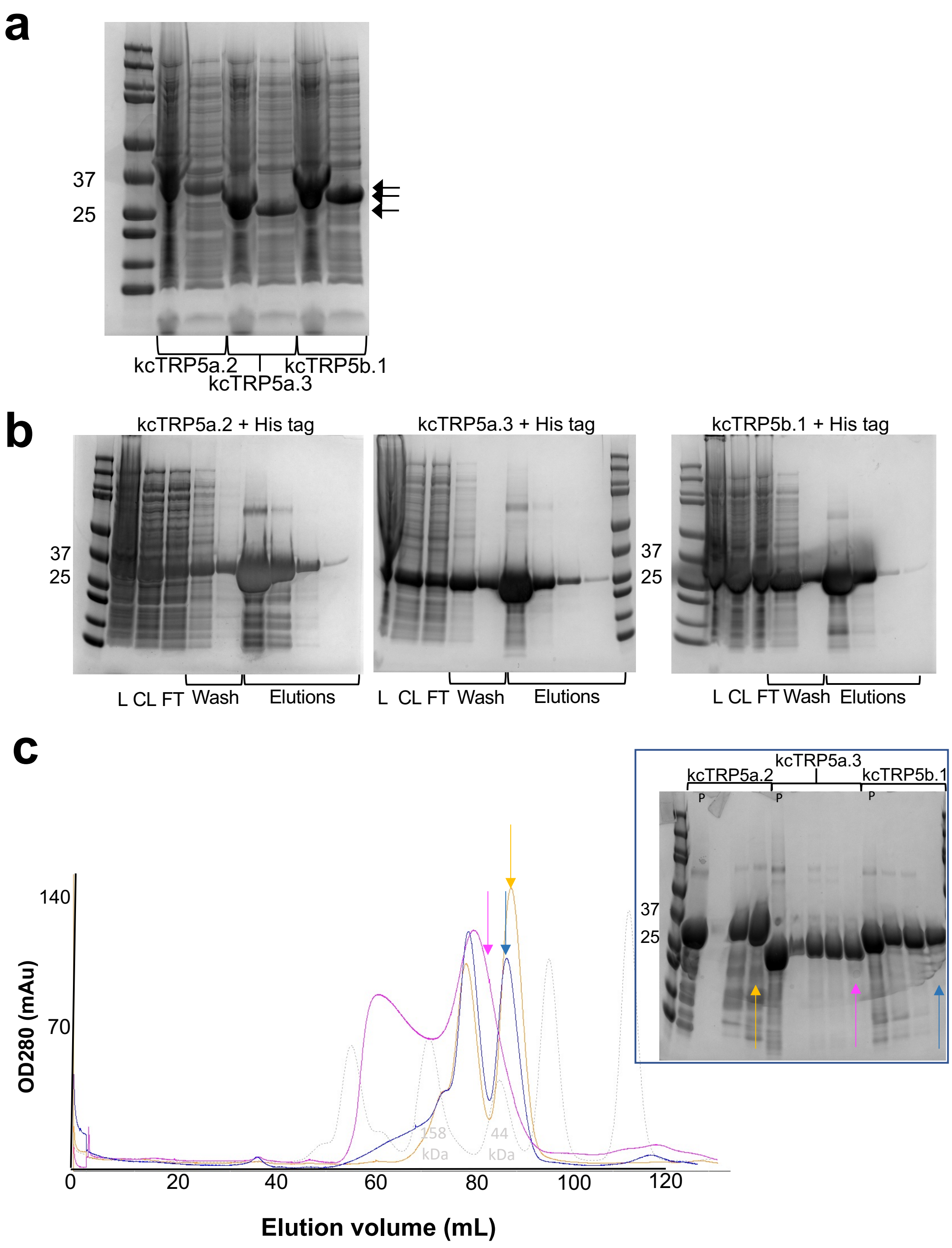

Supplementary Figure 10. Divergence in loop conformations and knotted topology between kcTRP5b.1 design model and crystal structure. (A) Original design (Gray) aligned to Crystallographic structure (rainbow) via residues 4-55 with Anti-bias 2Fo-Fc density (blue) and blue arrows indicating divergence. Anti-bias 2Fo-Fc map created from molecular replacement with original design followed by a round of refinement. No or poor density is observed between loop residue 48-60, 107-114, and 219-225 of the designed model. Strong density is observed between helices of observed protein fold. (B) Comparison of KnotProt plots between observed fold (upper) and original design (lower) show degeneration of knot from 5.1 (pentafoil) to 3.1 (trefoil).

Figure S9

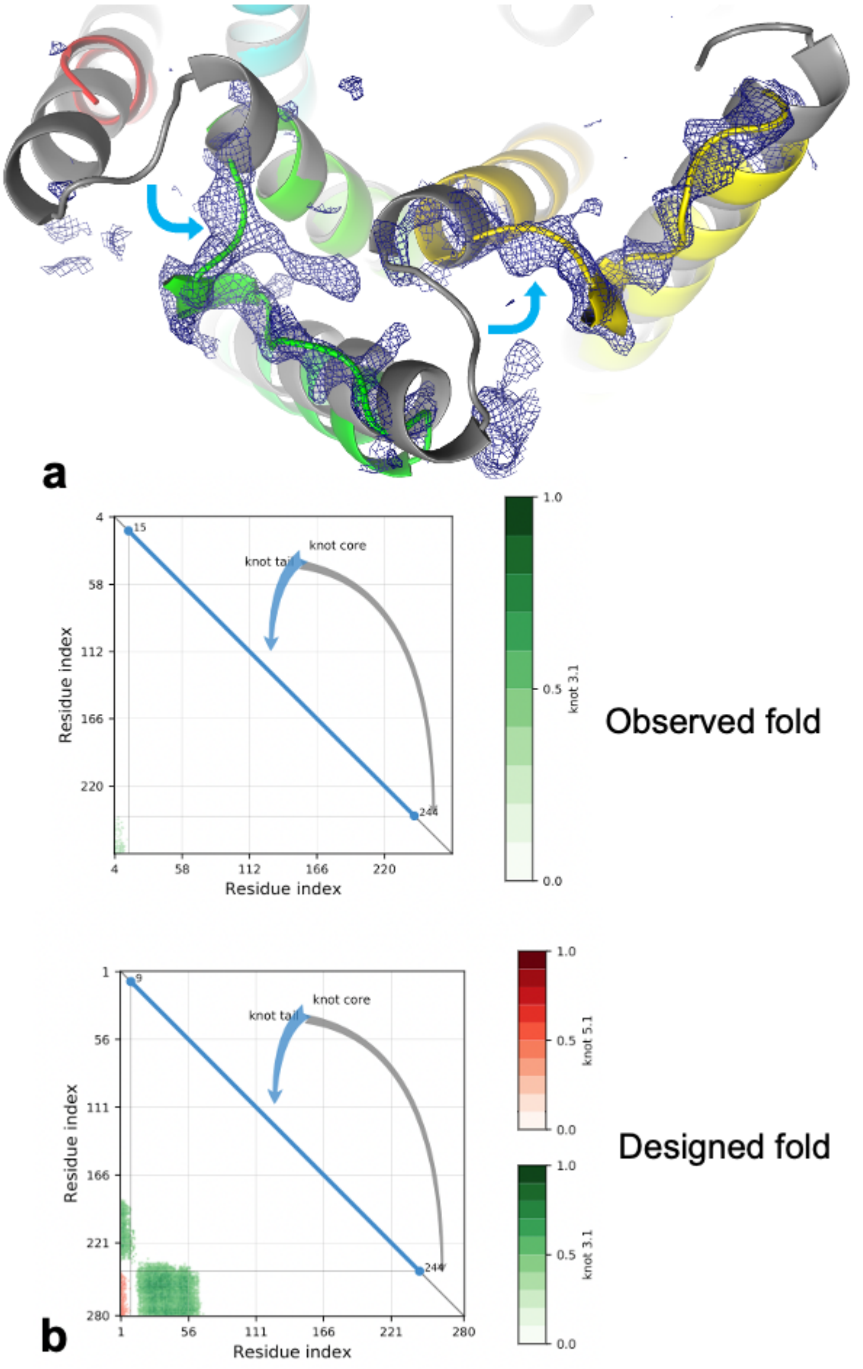

Supplementary Figure 11. Two-state analysis of the unfolding and refolding kinetics measured between 3.3 and 6.75 M GdmCl. Unfolding and refolding data measured between 3.3 and 6.75 M GdmCl was fitted to an equation describing a two-state kinetic model (left-hand equation). The values for $k_F^{H_2O}$ and $k_U^{H_2O}$ obtained from the fit were then used to calculate the Gibbs free energy difference between intermediate (I) and native (N) states, which was 22 kcal mol^{-1} and similar to that calculated from the stability measurements. The m_{k_F} and m_{k_U} values obtained from the fit were used to calculate the β-Tanford value (β_T) using the right-hand equation, and which was 0.53 indicating that over half of the solvent accessible surface area buried in folding from I to N, is already buried in the transition state. Source data are provided as a Source Data file.

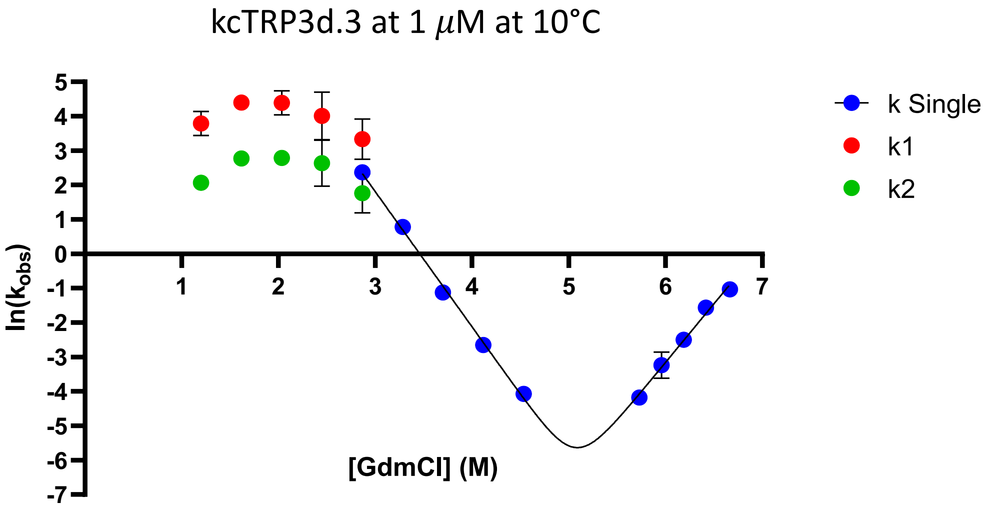

	mkf		ln_kf		mku		ln_ku	
	Value	SEM	Value	SEM	Value	SEM	Value	SEM
k Single	3.929	0.113	13.593	0.422	3.405	0.200	-23.591	1.242

$$\ln(k_{obs}) = \ln\left(k_F^{H_2O} e^{\left(-m_{k_F}[GdmCl]\right)} + k_U^{H_2O} e^{\left(m_{k_U}[GdmCl]\right)} \right)$$

$$\beta_T = \frac{m_{k_F}}{m_{k_U} + m_{k_F}}$$

Supplementary Figure 12. Evidence for and against three different folding pathways. ***Scheme I:*** Parallel pathways with an intermediate state on one pathway, and a small percentage of molecules folding along a fast track to the native state. ***Scheme II:*** Heterogeneity in the denatured state with folding proceeding through a common intermediate. ***Scheme III:*** Parallel refolding pathways on each of which there is an intermediate state.

Scheme I

Evidence for Scheme I

- Equilibrium results which show three states are populated: D, I and N
- Unfolding kinetics measure only k_U^3 which is rate-limiting on the pathway N $\rightarrow$ I $\rightarrow$ D . k_U^2 is very small therefore most molecules unfold via I.
- At low [den], predicts two folding phases, observe k_F^1 and k_F^2 (as under these conditions these are rate-limiting). Expect kinetic partitioning between the two pathways BUT can't test with amplitudes as the fluorescence change between D and I is not the same as between D and N (see equilibrium results).
- At higher [den], observe a single refolding phase corresponding to k_F^3., which becomes rate limiting on the D $\rightarrow$ I $\rightarrow$ N pathway.

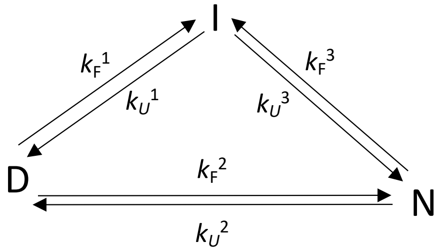

Evidence against Scheme I

- At higher [den], one would expect two refolding phases corresponding to k_F^3, which becomes rate limiting on the D $\rightarrow$ I $\rightarrow$ N pathway AND k_F^2

Scheme II

Evidence for Scheme II

- equilibrium results which show three states are populated: D, I and N (D_1 and D_2 are unlikely to be distinguishable by ensemble measurements using fluorescence.
- Unfolding kinetics measure only k_U^3 which is rate-limiting on the pathway N $\rightarrow$ I $\rightarrow$ D_1/D_2.
- At low [den], predicts two refolding rates observe k_F^1 and k_F^2 (as under these conditions these are rate-limiting).
- At higher [den], observe a single refolding phase corresponding to k_F^3., which becomes rate limiting on the D $\rightarrow$ I $\rightarrow$ N pathway.

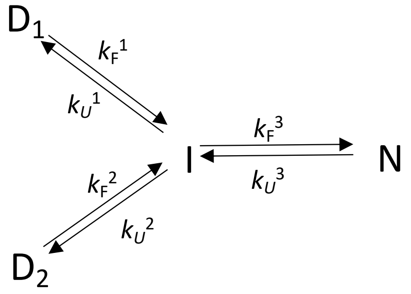

Evidence against Scheme II

- Expect kinetic partitioning between the two pathways such that the relative amplitudes of phases associated with k_F^1 and k_F^2 are the ratio of the rate constants. This isn't observed but can be explained if D_1 and D_2 have slightly different fluorescent properties.

Scheme III

Evidence for Scheme III

- Unfolding kinetics measure only k_U^3 which is rate-limiting on the pathway N $\rightarrow$ I_1 $\rightarrow$ D. The unfolding transition from N to I_2 is not observed as it may be too fast and not measurable in the stopped flow, or too slow, i.e, most molecules unfold via I_1 which is the energetically favourable pathway.
- At low [den], this scheme predicts two refolding rates observe k_F^1 and k_F^2 (as under these conditions these are rate-limiting).
- One would expect kinetic partioning of these two pathways which is not necessarily reflected by the relative amplitudes of the two refolding phases but this is easily resolved if I_1 and I_2 have slightly different properties.

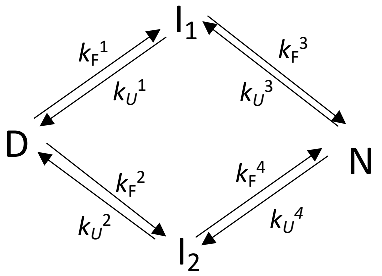

Evidence against Scheme III

- Equilibrium results show three not four states are populated. However, if the fluorescent properties, secondary structure and energetics of I_1 and I_2 are similar then two intermediate states would be difficult to resolve in the equilibrium experiments. There is precedence for this (see UCH-L1).
- At higher [den], this scheme would also predict two refolding phases, corresponding to k_F^3 and k_F^4 which become rate limiting on their respective pathways. Only one refolding phase is observed under these conditions, this can be explained by the I_2 $\rightarrow$ N transition having a much higher energy barrier, this folding may be too slow to observe (or most molecules fold via I_1).
